# *Leptospira* prevalence and lineages vary across land-use types due to shifts in small mammal communities

**DOI:** 10.1128/aem.02061-25

**Published:** 2026-01-30

**Authors:** Jeanne A. Rajaonarivelo, Kayla M. Kauffman, Toky M. Randriamoria, James P. Herrera, Natalie Wickenkamp, Magali Turpin, Fiona Baudino, Hillary S. Young, Voahangy Soarimalala, Steven M. Goodman, Charles L. Nunn, Pablo Tortosa

**Affiliations:** 1 Unité Mixte de Recherche Processus Infectieux en Milieu Insulaire Tropical (UMR PIMIT), Université de La Réunion, CNRS 9192, INSERM 1187, IRD 249, La Réunion, France; 2 Department of Ecology, Evolution and Marine Biology, University of California 8786, Santa Barbara, California, USA; 3 Association Vahatra, Antananarivo, Madagascar; 4 Duke Lemur Center SAVA Conservation, Duke University 3065, Durham, North Carolina, USA; 5 Department of Evolutionary Anthropology, Duke University 3065, Durham, North Carolina, USA; 6 Institut des Sciences et Techniques de l'Environnement, Université de Fianarantsoa 119026, Fianarantsoa, Madagascar; 7 Field Museum of Natural History 2415, Chicago, Illinois, USA; 8 Duke Global Health Institute, Duke University 3065, Durham, North Carolina, USA; University of Illinois Urbana-Champaign, Urbana, Illinois, USA

**Keywords:** land cover change, emerging neglected tropical disease, invasive species, zoonosis, wildlife, One Health

## Abstract

Human-induced land-use change can affect the composition of small mammal communities and the ecology of their zoonotic pathogens — yet questions remain on the direction and generality of these changes, which can have opposite effects on disease prevalence depending on the ecological context and pathogen involved. These contrasting patterns highlight the need to investigate how specific host-pathogen assemblages respond to local anthropogenic land-use mosaics. To address this need, we studied terrestrial and bat species composition, *Leptospira* infection prevalence, and *Leptospira* species composition across a mosaic of land-use types in northeastern Madagascar. We found differences in host communities between forested, agricultural, and village land-use types for both bat (*n* = 400) and terrestrial (*n* = 2,053) small mammal communities. *Leptospira* infection prevalence was higher in bats (37.7%) than in terrestrial small mammals (13.8%), and bats were infected with *Leptospira* strains that were molecularly distinct from those shed by terrestrial small mammals. Non-native mice and rats were almost exclusively infected with cosmopolitan *L. kirschneri* and *L. interrogans,* respectively, while some native terrestrial small mammals sheltered *L. mayottensis,* and bats hosted a more diverse set of *Leptospira* species. *Leptospira* prevalence across land-use types varied in terrestrial small mammals, but not in bats. Altogether, the highest prevalence occurred in mice in flooded rice fields. Our data show that land use predominantly impacts *Leptospira* infecting terrestrial mammals, likely due to habitat disturbance favoring replacement of endemic hosts and pathogens with Muridae rodents and their associated pathogens, many of which are zoonotic.

**IMPORTANCE:**

Leptospirosis, a globally distributed, environmentally transmitted zoonosis, causes 2.9 million disability-adjusted life years annually, primarily among rural farmers in tropical regions. Infected animals’ urine contaminates soils and water with *Leptospira* bacteria, where other individuals are then exposed. Understanding the impact of land use on the transmission of this disease is of considerable importance. In Madagascar, infection dynamics are impacted by the combined effects of converting forests to agricultural fields and colonization of these areas by non-native mammal species, which carry molecularly distinct lineages of *Leptospira*. We show that land use corresponds to the replacement of native species and endemic *Leptospira* lineages with non-native species and their cosmopolitan *L. interrogans* and *L. kirschneri*. Together, this contributes to higher infection prevalence in more disturbed habitats like flooded rice fields, where >50% of mice captured were infected, highlighting the important effects of land use on *Leptospira* prevalence and presence, which together impact zoonotic risk.

## INTRODUCTION

Land-use change is considered a major driver of disease emergence and re-emergence globally (1–5). As natural habitats are converted into agricultural, anthropogenic systems, the species that rely on those natural habitats tend to decline. In contrast, synanthropic species that thrive in anthropogenic habitats tend to become more abundant (6, 7). The same mechanisms that allow these species to thrive in human-dominated landscapes — fast life histories, high densities, and being both diet and habitat generalists — also make them more likely to carry and transmit a wider range of parasites (8–10). Thus, the habitat-induced shifts in the host community are also altering the parasite community and potentially allowing for novel host-parasite associations (6, 11). Furthermore, synanthropic animals found in more disturbed habitats are more likely to harbor zoonotic parasites and transmit them to people due to their proximity and thus increased direct and indirect contact with people (7, 12, 13). Thus, it is vital to study how land-use change impacts host community composition and, in turn, impacts which parasites are present, their prevalence, and their host associations.

In reality, however, the disease-land use relationship is neither linear nor consistent across disease systems (1, 14). For example, the level of disturbance and habitat preferences of competent hosts result in Lyme prevalence often being highest at intermediate levels of disturbance, while plague prevalence in animals is highest at intermediate to high levels of disturbance (15, 16). The mechanisms for these changes are also varied: land-use change can alter environmental conditions to bring novel host species into contact (e.g., Nipah [17]), favor different vector communities (e.g., leishmaniosis [5, 18]), alter pathogen survival in the environment (e.g., *Leptospira* [19, 20]), and facilitate parasite invasions (e.g., *Trypanosoma* [21]). While some of these changes likely have generalizable patterns (e.g., all forms of land-use change likely tend to bring novel host species into contact), others will be idiosyncratic to the pathogen and type of land-use change (e.g., environmental changes to rice might be conducive to a waterborne pathogen while conversion to corn would not increase that pathogen). Predicting the impacts of land-use change on host-parasite-environment interactions often requires disentangling the effects of host community composition and environmental conditions on the transmission of a given parasite across land-use types (3).

The impact of land-use change on disease transmission is highly relevant in Madagascar, an island nation with high levels of species diversity, numerous zoonotic pathogens of public health relevance, extensive poverty, and limited access to healthcare (22–24). As a biodiversity hotspot, many animals native to Madagascar are endemic (i.e., occur nowhere else in the world), and little is known about the pathogens they carry. Native non-primate small mammals include 46 species of bats, 31 endemic species of tenrecs (Tenrecidae), and 28 endemic species of rodents (Nesomyidae, subfamily Nesomyinae) (25). Non-native (introduced) small mammal species in Madagascar include Muridae rodents (synanthropic mice and rats), which favor anthropogenic habitats and are well-known carriers of zoonoses (25, 26). Notable infectious agents with known or inferred zoonotic potential on Madagascar include hantaviruses (27, 28), astroviruses (29, 30), paramyxoviruses (31, 32), *Yersinia pestis,* the causative agent of plague (16, 33), enterotoxigenic *Escherichia coli* and *Shigella,* which are both responsible for diarrheal diseases (34, 35), and *Leptospira*, which causes leptospirosis (36, 37). These zoonotic pathogens are a public health challenge in many areas of the country, and the majority of these are primarily associated with non-native rodents as compared to native species (32). The transition from communities of predominantly native hosts with their endemic parasites to communities of predominantly non-native hosts and their cosmopolitan parasites makes Madagascar an ideal location for investigating host and parasite community composition shifts and novel host-parasite associations.

Madagascar is also experiencing dramatic land-use conversion. Rapid deforestation over the last 60 years has resulted in the island losing around 44% of its native forest (38). Nearly 80% of the Malagasy people live in rural areas, and the majority (82%) live below the poverty line and rely on natural resources and agriculture for subsistence (39, 40). Forests are transformed for the cultivation of rice, cassava, and other subsistence crops, particularly in the eastern portion of the country (41). Madagascar produces cash crops that are in high demand globally, including cloves, coffee, cocoa, and lychee, and is the world’s largest producer of vanilla (42–44), which is a significant source of income for farmers in the northeast (45, 46). Increases in demand for cash crops and soil depletion associated with hillside agricultural practices have accelerated the expansion of cultivation, leading to further forest fragmentation and transformation (47, 48). The combination of increased rates of land-use change, transitioning communities of mammal species and their associated pathogens, and close contact between humans and natural environments makes Madagascar an important location to address questions on how land-use conversion alters host and parasite communities and thus the ecology of infectious diseases (49).

*Leptospira* is an excellent organism for exploring how land use impacts pathogen dynamics due to environmental and host community composition changes. The different species and serovars of *Leptospira* tend to be associated with different sets of host species; however, the bacterium is only weakly host specific — one host species can be infected with multiple types of *Leptospira,* and one type of *Leptospira* can infect multiple host species. In Madagascar, *L. mayottensis,* alongside numerous other unnamed lineages, suggests the bacterium has undergone long-term coevolution with native small mammal hosts (50–54) and that *L. mayottensis* almost certainly originated on Madagascar (55). Three other lineages of *Leptospira* (*interrogans, kirschneri,* and *borgpetersenii*) are introduced and have undergone varying degrees of divergence, particularly in bats (25, 36, 50, 51, 53, 56, 57). These lineages can be broadly grouped into cosmopolitan (i.e., found worldwide) species (*L. interrogans* and *L. kirschneri*) and endemic species (*L. borgpetersenii* and *L. mayottensis*). The cosmopolitan species are generally found in Muridae rodents (36, 50, 51, 57), while the endemic species are primarily found in bats, tenrecs, and native rodents (53, 57).

In northeast Madagascar, *Leptospira* prevalence was higher in small mammals trapped in anthropogenic habitats where introduced rodents were more abundant than in native forested habitats (51). Anthropogenic land use promotes the colonization of introduced rats and mice, which harbor the cosmopolitan strains of *L. interrogans* and *L. kirschneri*, respectively, and consequently may modify local infection patterns (51, 58). Therefore, this is a model system to explore whether land use induces animal community composition changes that increase parasite prevalence and whether these host community changes result in different host-*Leptospira* species associations (51, 52, 59).

*Leptospira* is transmitted indirectly between animals and people through contaminated water and soil (60). Broadly, farmers in tropical regions are at high risk for infection, particularly those who use flooded rice farming techniques, such as those used in Madagascar (61). In Madagascar, *Leptospira* has been found in people, native and non-native wild animals, bats, cows, and pigs (25, 50, 56, 62–64). However, the epidemiologic data on *Leptospira* in people are limited; a single study reported a 2.9% (20/678) seroprevalence in people with serovars belonging to *L. interrogans* and *L. noguchii* (65). Clinical surveys carried out on neighboring southwestern Indian Ocean islands, as well as experimental studies, have brought insightful information on the virulence of *Leptospira* isolated from rats, tenrecs, and bats. Indeed, although *L. mayottensis* and *borgpetersenii* are zoonotic on Mayotte Island (Comoros Archipelago), their virulence is significantly lower than that of rat-borne *L. interrogans* (66, 67). Similarly, minimal clinical signs were found in experimental infection of hamsters, a model of acute infections in humans, with *L. borgpetersenii* isolated from Malagasy (68). Overall, this suggests that endemic species of *Leptospira* may be less pathogenic in people than cosmopolitan species and that land-use change may favor zoonotic lineages.

Across a mosaic of anthropogenic disturbance in and around Marojejy National Park in northeastern Madagascar (25), we investigated how land use corresponds with the composition of host communities, *Leptospira* infection prevalence, and *Leptospira*-host species associations in terrestrial small mammals and bats. Expanding on previous work on Malagasy small mammals at the same site (51), we used samples from additional terrestrial small mammals collected over an additional 3 years (in total 5 years) and bats (only during the added 3 years) across multiple land-use types. From these extensive host surveys, we estimated the composition, diversity, and relative abundance of terrestrial small mammal and bat hosts across the habitat mosaic to assess the differences in *Leptospira* infection prevalence among land-use types. Additionally, we used extensive sequencing data from animals infected with *Leptospira* to understand how the composition of *Leptospira* varied across hosts and habitats.

Using both host and pathogen data, we investigated the following questions: (i) How does the habitat type influence the species composition and density of terrestrial small mammals and bats? We hypothesized that native host species prefer forested or less disturbed habitat types, while synanthropic non-native species would occur at highest abundance in non-native habitats close to human settlements. (ii) How do host composition and environmental factors associated with land-use change impact *Leptospira* prevalence? Following previous findings (51), we hypothesized that prevalence would be highest in highly disturbed areas favored by non-native small mammal species, such as villages and exposed warm, wet areas such as rice fields, where *Leptospira* persists in the environment. (iii) Which lineages of *Leptospira* are found in which hosts and habitat types? This last question allows us to identify habitat types where pathogen switches have likely occurred. Based on previous reports of host-pathogen specificity, we hypothesized that bats and small mammals host different lineages of *Leptospira*. However, in places where a given host species was less abundant, we anticipated finding lineages typically associated with more abundant host species. Cumulatively, our study provides insights into the mechanisms by which land-use change may alter *Leptospira* risk by investigating associations between environment, small mammal community composition, and *Leptospira* lineages across a land-use mosaic.

## RESULTS

### Small mammal communities across land-use types

#### Terrestrial small mammals

**Fig 1:**
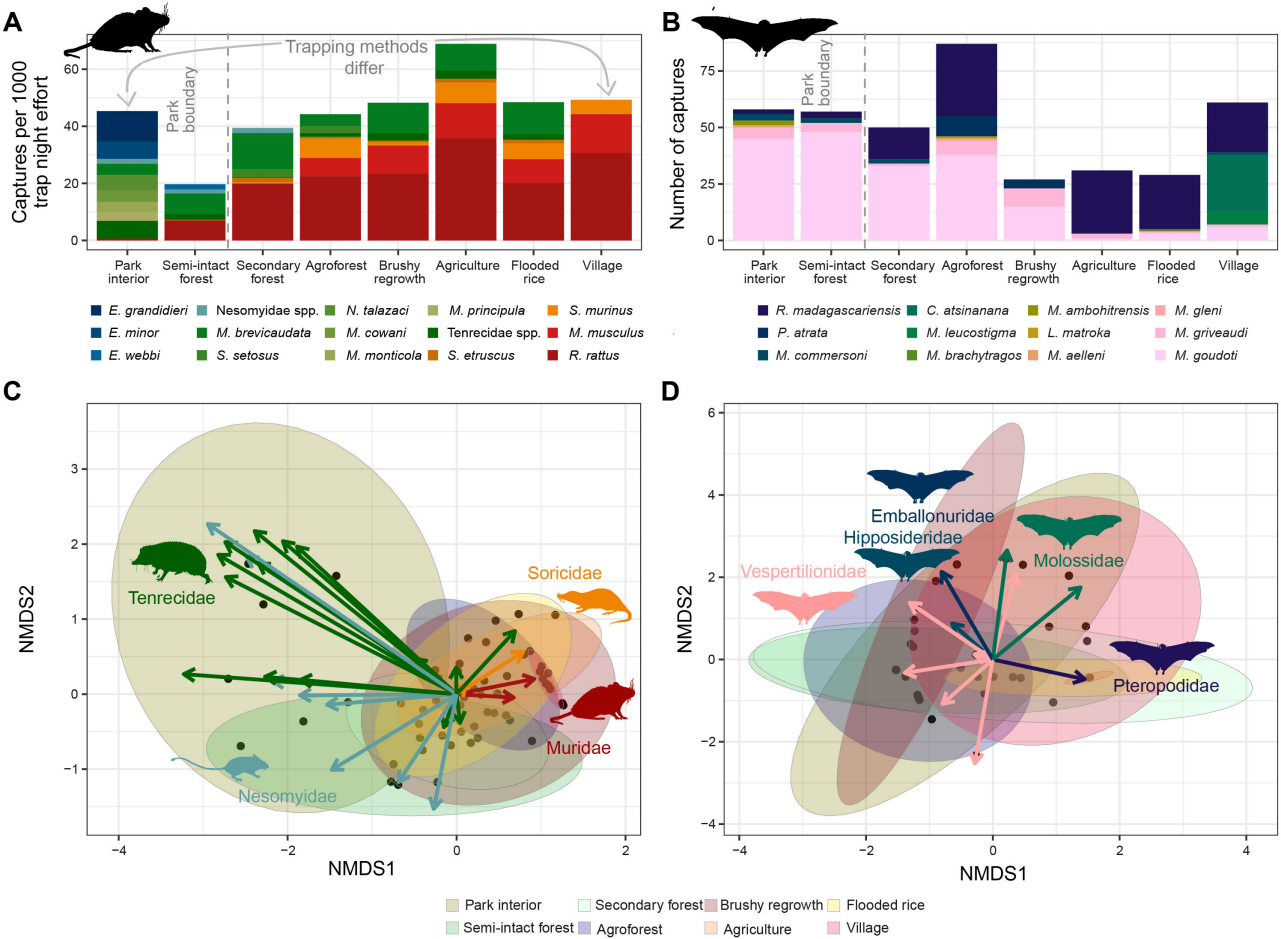
(**A**) Terrestrial small mammal composition transitioned from predominantly native Nesomyidae and Tenrecidae communities, shown in blues and greens inside the park interior, to predominantly non-native Muridae communities on the park exterior. For visual clarity, species with less than 10 individuals captured were grouped by family (see Fig. S2 for all species). (**B**) Bat species composition also shifted from Vespertilionidae communities in forested areas, Pteropodidae (*Rousettus madagascariensis*) in agricultural areas, rice fields and villages, and Molossidae communities around the homes in the villages. The two-dimensional non-metric multidimensional scaling (NMDS) plots display the pronounced separation of terrestrial mammals (**C**) between forested and non-forested habitat types and the lack of this contrast among bats (**D**). The ellipses in the NMDS plots represent the 95% confidence intervals of the total population by habitat type (t-distributions), and the arrows indicate the strength and direction of the linear correlation between each species and the ordination scores. The arrows are colored by family (see Fig. S3 for plots with arrows labeled by species).

From 2017 to 2021, 2,053 terrestrial small mammals representing 28 species were captured, including several species endemic to Madagascar from two groups: tenrecs from the Tenrecidae family (*Microgale* spp., *Nesogale* spp., *Oryzorictes hova*, *Setifer setosus*, and *Tenrec ecaudatus*) and native Nesomyinae rodents (*Eliurus* spp., *Nesomys* spp., and *Voalavo gymnocaudus*; Fig. 1A; Table S1). However, the majority (*n* = 1,354; 65.9%) of captured animals were non-native Muridae rodents (*Rattus rattus* and *Mus musculus*) and Soricidae shrews (*Suncus murinus* and *S. etruscus*). *Rattus* were captured in all habitat types, including inside the park; they were the most common species captured (*n* = 898; 43.7%), followed by *Microgale brevicaudata* (*n* = 366; 17.8%), introduced mice (*n* = 273; 13.3%) and shrews (*n* = 183; 8.9%). *Mus* were trapped in anthropogenic habitats and absent from forest habitats.

Across habitat types, we found significant differences in the proportion of captures of native species (ANOVA: F = 20.25, df = 7, *P* < 0.001), animal density (number captured/trap effort; ANOVA: F = 5.05, df = 7, *P* < 0.001), species richness (ANOVA: F = 6.56, df = 7, *P* < 0.001), and Shannon diversity (ANOVA: F = 5.03, df = 7, *P* < 0.001), while species evenness did not differ significantly across habitat types (ANOVA: F = 0.784, df = 7, *P* = 0.604). Animal density tended to be lower in the semi-intact forest (Fig. S1, Table S2). Species richness was significantly higher in the park interior than in all other habitat types (Tukey post hoc test: *P* < 0.05; Table S2) except agroforest (*P* = 0.16) and agriculture (*P* = 0.28), but these differences only resulted in higher Shannon’s diversity in the park compared to the secondary forest (*P* = 0.01), brushy regrowth (*P* = 0.03), and village (*P* < 0.001; Table S2). The proportion of native species was significantly higher in forested habitats inside the park than outside, and lower in the village than in the secondary forest (Fig. S1, Table S2). The trapping methods limited our ability to make comparisons between the village, where traps were placed inside people’s homes, and the park interior, where transects and a greater ratio of pitfall traps to Sherman/National traps were used, and all other habitat types, where uniform trap grids and pitfall lines were used (see Methods). Notably, most tenrecs (94.0%, 691/735) were captured in pitfall traps.

These community differences result in a pronounced separation in two-dimensional NMDS ordination space (Fig. 1C), with tenrec and endemic rodent species indicating trap grids located in the park interior and less disturbed forests, and introduced Muridae and Soricidae species indicating habitat types outside of the park. The semi-intact forest, located just inside the national park, bridged the differences between inside and outside the park due to the presence of two tenrec species (*Microgale brevicaudata* and *Setifer setosus*), which were captured both in forests and in cultivated fields outside forests, and by the presence of two endemic rodents (*Eliurus webbi* and *Nesomys audeberti*). The animals captured in each habitat varied in both species composition (PERMANOVA: F = 5.06, df = 7, *P* < 0.001) and variability (PERMDIST: F = 2.92, df = 7, *P* = 0.012).

#### Bats

From 2019 to 2021, 400 bats representing 12 native species (Fig. 1B; Table S2) were captured, including Molossidae (*Chaerephon atsinanana* and *Mops leucostigma*), Vespertilionidae (*Laephotis matroka* and *Myotis goudoti*), Hipposideridae (*Macronycteris commersoni*), Emballonuridae (*Paremballonura atrata*), and Pteropodidae (*Rousettus madagascariensis*). Though native, none of these species is endemic to Madagascar. *Myotis goudoti* (*n* = 189; 47.2%) was the most captured species and was found in all habitat types, though more frequently in forested habitats. *Rousettus madagascariensis*, the second most abundant species (*n* = 125; 31,2%), was primarily captured in agricultural fields and in the village.

Compared to terrestrial small mammals, the distribution of species composition in bat communities varied less between land-use types (Fig. 1D; Fig. S1). We found no significant variation in the number of animals captured (ANOVA: F = 0.81, df = 7, *P* = 0.57), species evenness (Kruskal-Wallis: χ^2^ = 1.76, df = 7, *P* = 0.97), species richness (Kruskal-Wallis: χ^2^ = 3.47, df = 7, *P* = 0.84), and Shannon’s diversity index (Kruskal-Wallis: χ^2^ = 2.27, df = 7, *P* = 0.94) between habitat types. However, we did observe significant differences in communities by habitat type in NMDS space (Fig. 1D). These differences were due to compositional differences (PERMANOVA: F = 2.80, df = 7, *P* < 0.001) and not variability (PERMDIST: F = 1.78, df = 7, *P* = 0.109).

### *Leptospira* infection prevalence across land-use types

#### Terrestrial small mammals

Out of 2,053 captured terrestrial small mammals, 2,032 individuals were tested, and 280 were qPCR positive, leading to an overall infection prevalence of 13.8%. Most infected terrestrial animals, 86.1% (*n* = 241), were introduced species, while native species represented only 13.9% of the positive cases (*n* = 39; Table S1). The most infected host species was *Mus musculus* (100 qPCR positive/272 individuals tested; 36.8%), followed by *Rattus rattus* (125/896; 13.9%), *Suncus* spp. (14/177; 7.9%)*, Setifer setosus* (3/54; 5.5%), and *Microgale brevicaudata* (20/333; 6.0%). No infected individuals were found in several endemic species; however, few individuals of these species were captured (Table S1; range 1 to 31). Assuming a population prevalence equal to what we observed in our abundant native species (5.0%), a sample size of 73 individuals per species would be needed to determine that no individuals of a particular species were infected (power 80%, α = 0.05).

**Fig 2:**
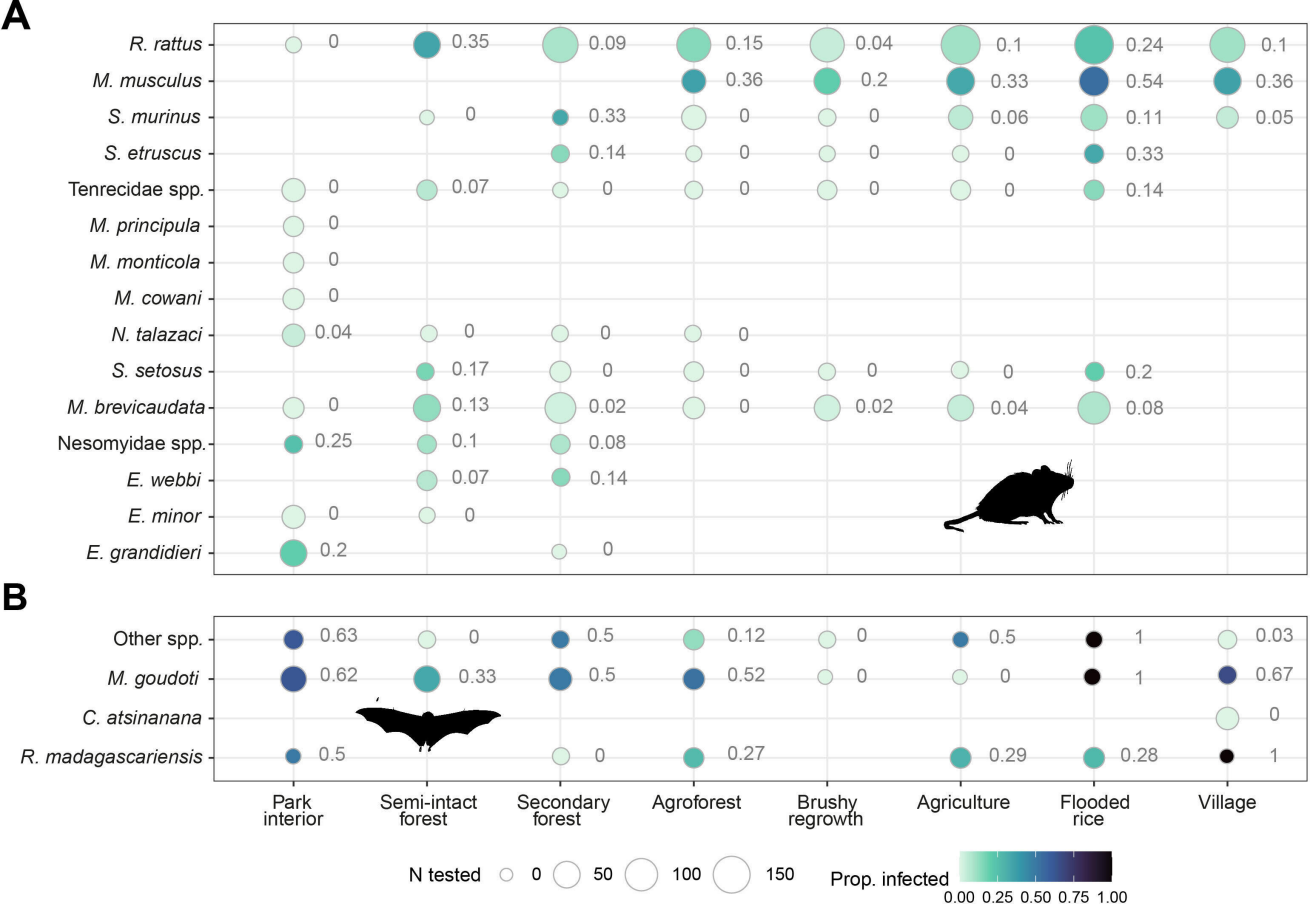
The proportion of infected (**A**) terrestrial small mammals and (**B**) bats by species and habitat type. The size of the circles represents the number of animals tested, and the adjacent numbers and shades of the circles represent the proportion of those animals that were infected. Species with fewer than 10 individuals tested across all habitat types were grouped by family (terrestrial small mammals) or as “other species” (bats). See Table S1 for the complete results of captures and testing in all species.

The proportion of infected individuals (prevalence herein) was highest in the rice fields, semi-intact forest, and village homes, and lowest in the park interior, secondary forest, and agricultural areas (Fig. 2A; Table S1). The majority of mice captured in flooded rice fields were infected (40/74; 54%). *Rattus rattus* and *M. brevicaudata* were more commonly infected in the semi-intact forest, and shrews (*Suncus murinu*s) were more commonly infected in the secondary forest.

**Fig 3:**
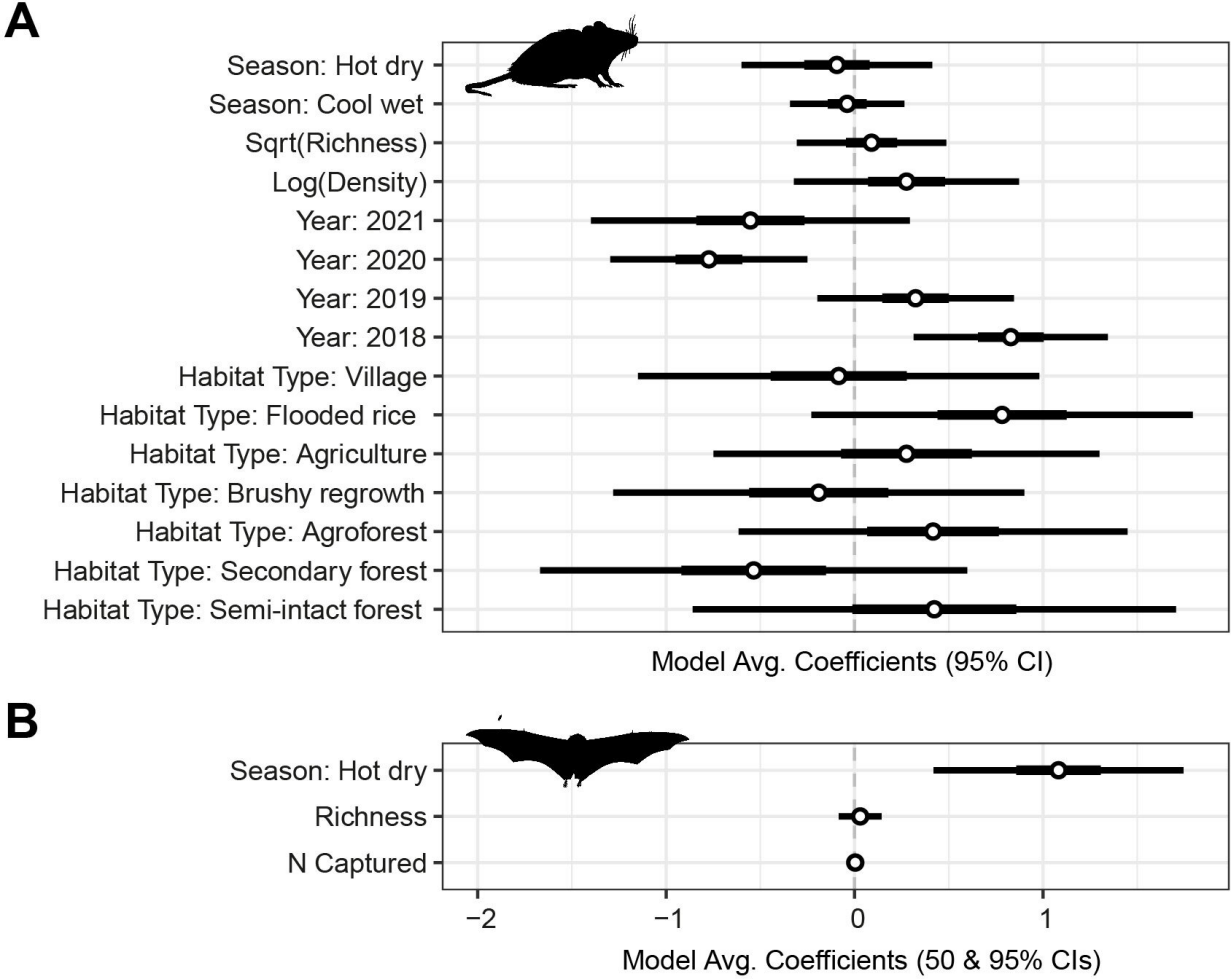
Full model-averaged coefficients plot for infection probability in (**A**) terrestrial small mammals and (**B**) bats. Bars show the 50% and 95% confidence intervals for the estimated coefficients. Reference variables were set as follows: 2017 (year for terrestrial small mammals), warm wet (season), and park interior (habitat type). See Table S3 for numeric estimates.

Binomial models of the number of infected animals of those tested during each trapping period were used to consider the joint effects of species composition and habitat type while accounting for seasonal and interannual variability. The most important predictors of infection were habitat type and year, followed by log animal density, square-root species richness, and season (AICc sum weights [sw] = 1.0, 1.0, 0.56, 0.22, 0.15; Table S3). In agreement with the habitat-wise estimates of prevalence, the log odds of infection for terrestrial small mammals were highest in flooded rice fields (β = 0.78 ± 0.52) and semi-intact forest (β = 0.42 ± 0.65), and lowest in the secondary forest (β = −0.54 ± 0.58; Fig. 3A; Table S3). Large interannual differences were found between terrestrial small mammals captured in 2018 and 2019 and those captured in 2017, 2020, and 2021. Samples in the 2018-2019 period had higher odds of infection than in the other years. Due to the study design, disentangling the effects of study locality and interannual variability (year) was not possible, as localities were sampled sequentially, not simultaneously. Both species richness (β = 0.09 ± 0.2) and density (log number of captured terrestrial mammals per night’s trap effort [β = 0.28 ± 0.3]) were weakly positively associated with the log odds of infection. Though the effects of seasonality were weak, infection odds were slightly higher in the warm wet season (reference category) than in both other seasons (β_cool,wet_ = −0.04 ± 0.34; β_hot,dry_ = −0.09 ± 0.26).

#### Bats

Infection prevalence in bats (37.7%; 106 qPCR positive/281 individuals tested) was significantly higher than in terrestrial small mammals (Kruskal-Wallis: χ^2^ = 100.07, df = 1, *P* <0.001). The most abundant bat species, *Myotis goudoti,* displayed the highest prevalence (76/153; 49.7%), which was much greater than that of the second most abundant species, *Rousettus madagascariensis* (16/58; 27.6%). We identified infected individuals of all but one species of bat (*n* = 7) in the family Vespertilionidae, and no infected Molossidae (*n* = 25) or Emballonuridae (*n* = 13) individuals, despite having sufficient power to do so, if infection prevalence was similar to what we observed in *R. madagascariensis* (sample size =9, power 80%, α = 0.05).

We identified infected bats captured in all habitat types except brushy regrowth, with the highest proportion of infected animals found in the park interior (36/58; 62.1%), secondary forest (15/35; 42.9%), and rice fields (10/23; 43.5%; Fig. 2B; Table S1). In general, the variability in infection prevalence between habitat types was greater in terrestrial small mammals than in bats. However, similar to terrestrial small mammals, no linear trends with the level of disturbance were observed (Fig. 2B). Binomial models of the number of infected bats of those tested during each trapping period revealed that habitat type was not important to the overall odds of infection in bats; however, season (sw = 1), species richness (sw = 0.33), and the number of animals captured (sw = 0.28) were important (Table S3). Because bats were trapped over fewer periods than terrestrial small mammals, season and year were aliased (trapping only occurred in the hot, dry period of 2019). Thus, only the season was considered in the bat model. Contrary to the terrestrial mammals, we found a strong seasonal effect in infection odds in bats, which were more likely to be infected during the hot dry season than the warm wet season (β = 1.08 ± 0.34; Fig. 3B; Table S3). However, due to the schedule of trapping bats, we cannot distinguish between season and location-level effects. Again, both species richness (β = 0.03 ± 0.06) and density (number of captured bats [β = 0.00 ± 0.01]) were weakly positively associated with the log odds of infection.

### *Leptospira* lineages by host species and land-use type

**Fig 4:**
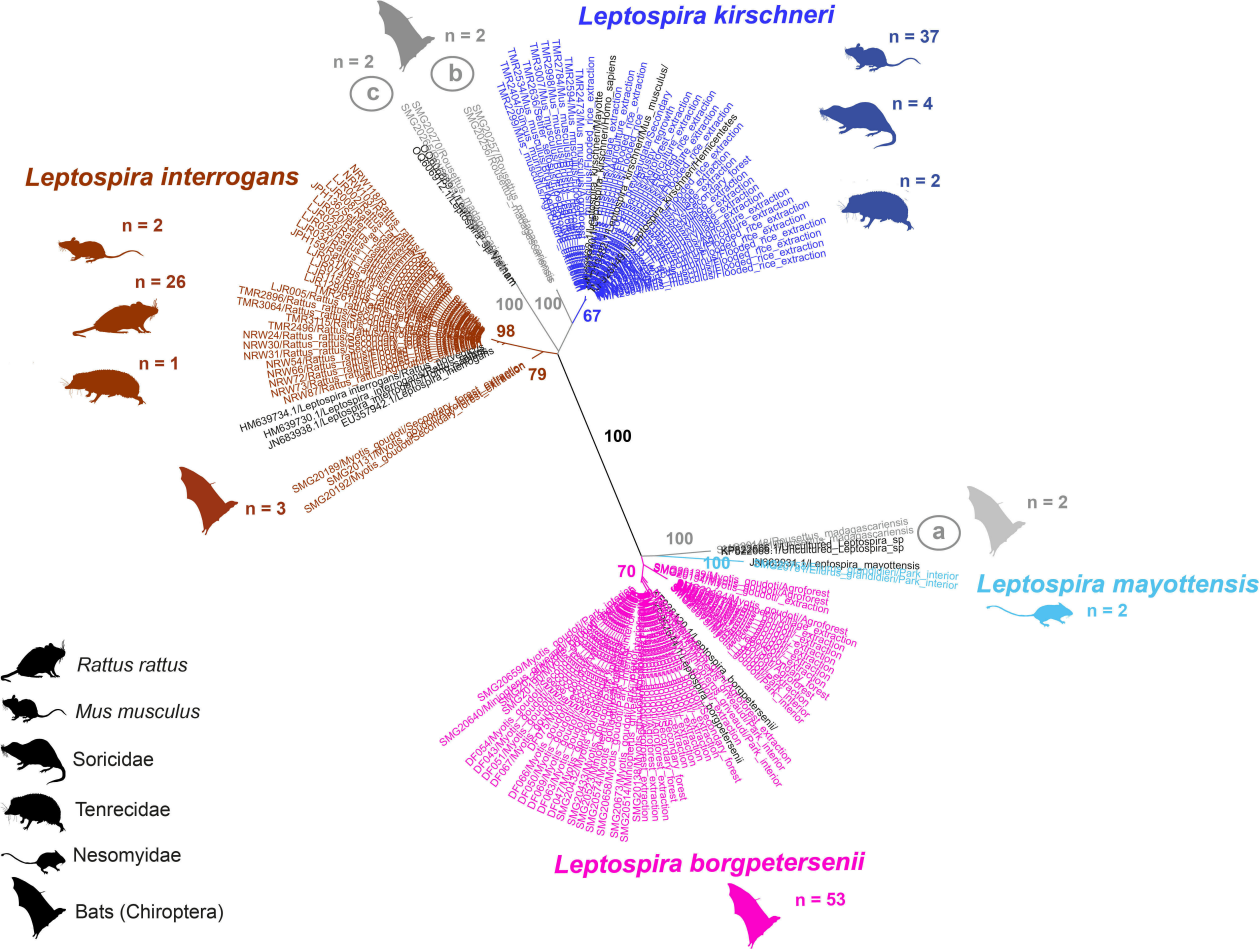
Maximum-likelihood (ML) radial phylogenetic tree built with 136 *Leptospira secY* sequences from small mammal species trapped in northeast Madagascar and 15 reference sequences (in black). The numbers represent the bootstrap percentage from 1,000 replicates and are indicated when above 50%.

**Fig 5:**
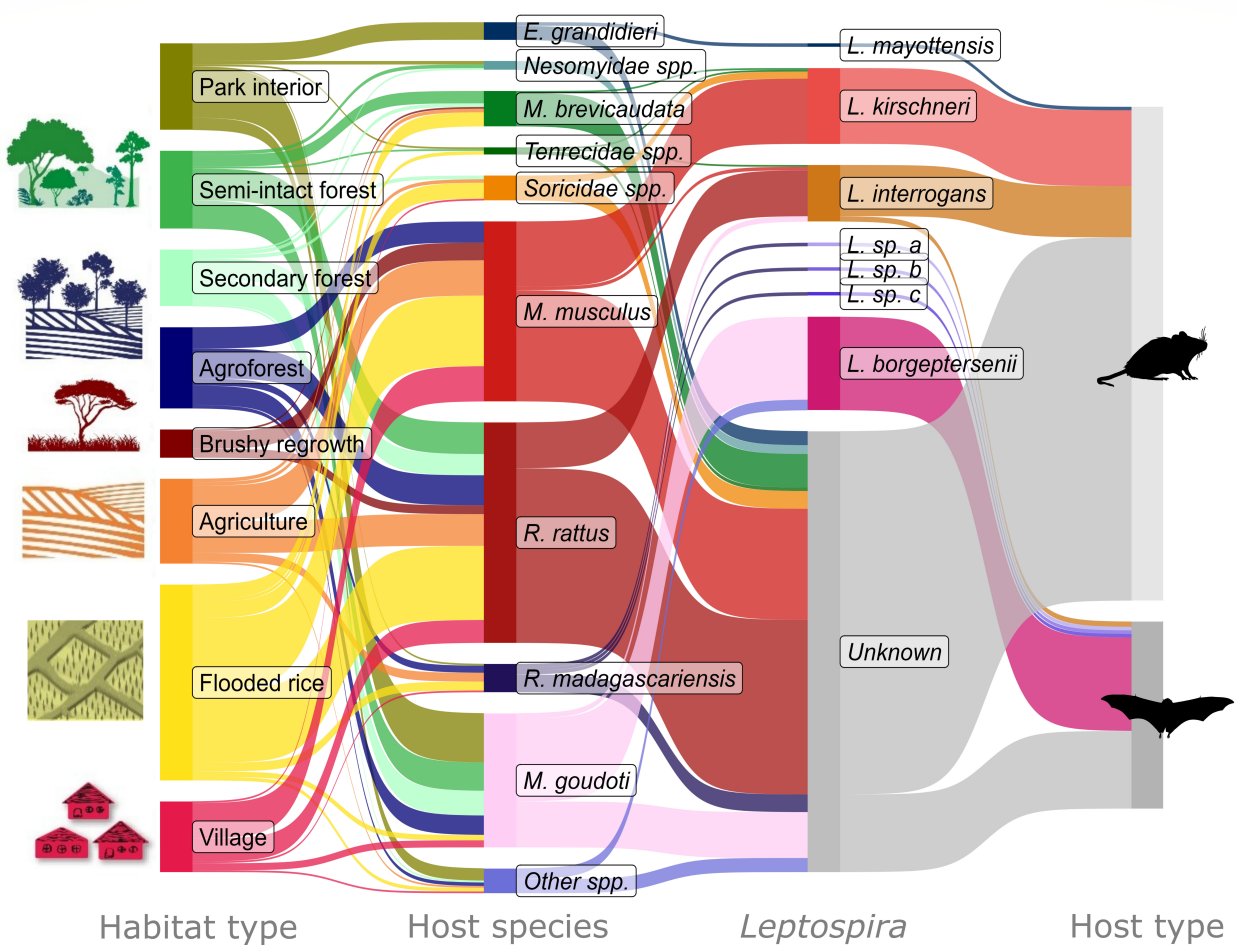
Sankey diagram showing the connections between habitat type, animal host species, and *Leptospira* species. The flows indicate the number of individuals connecting each habitat, host, infection, and host type. Samples that were positive but were not successfully sequenced are indicated as “Unknown.” Only *Leptospira*-positive animals are included in this figure, and species with fewer than 10 infected individuals are grouped by family (terrestrial mammals) or as “Other species” (bats). All species and all captured animals are shown in interactive Fig. S4. The diagram was created using the “ggsankey” package (69).

We successfully sequenced the *secY* gene of 136 of our 380 positive samples, 62 of which were from bats and 74 from terrestrial small mammals. Phylogenetic analysis identified *Leptospira* at the species level and showed a division into several distinct genetic clades (Fig. 4) primarily along *Leptospira* species lines, with a few additional subgroups (a–c). The separation between host species, habitat type, and *Leptospira* lineage revealed that some *Leptospira* species were classified as “cosmopolitan” or associated with non-native species and disturbed habitats, and others as “forest-associated” or found in forest habitats and native species (Fig. 5; Fig. S2, Table S4).

The three clades that corresponded with endemic *Leptospira* lineages were primarily found in bats and native species of terrestrial small mammals. All the sequences clustered with the *L. borgpetersenii* reference sequences were from Malagasy bats (Fig. 4). The majority of *L. borgpetersenii* sequences corresponded to the haplotype closely related to *secY^3^_48_* and were found in *Myotis* (*n* = 45) and *Miniopterus* (*n* = 6) species. The remaining two sequences of *L. borgpetersenii* were assigned to a haplotype close to *secY^3^_61_* and found in *Myotis* species. Most of these individuals were trapped in forest habitats, but a few individuals trapped in the rice fields (*n* = 3) and village (*n* = 3) were also infected with this clade of *Leptospira* (Fig. 5). Both of the sequences in the *L. mayottensis* cluster were from endemic rodents (*Eliurus grandidieri*) captured inside the national park (Fig. 4 and 5). The haplotype of these samples was closely related to *secY^3^_56_*. The final endemic clade (herein referred to as *Leptospira* species *a*) was closely related to *L. mayottensis*. The two specimens forming this clade were obtained from *Rousettus madagascariensis*.

The four “cosmopolitan” clades consisted of *L. interrogans, L. kirschneri,* and two novel groups (*L. sp. b* and *L. sp. c*). The sequences forming the clade corresponding to reference *L. interrogans* were from terrestrial mammals (*n* = 29) and *M. goudoti* bats (*n* = 3) (Fig. 4). The haplotype closely related to the *secY^3^_13_* was found in all *R. rattus* (*n* = 26) which were mostly trapped in agricultural fields, in one *M. musculus* trapped in the village and another captured from the flooded rice, and in a single tenrec (*Setifer setosus*) captured in flooded rice. The bat sequences in this clade were from two *M. goudoti* captured in the secondary forest and another captured in the agroforest (Fig. 5). These bat-associated sequences were closely related to the *secY^3^_9_* haplotype, forming a distinct subgroup (Fig. 4).

All sequences in the clade associated with *L. kirschneri* reference sequences were assigned to a single *secY^3^_22_* allele. Most of the samples in this group were *M. musculus* (*n* = 37). However, samples from one tenrec (*S. setosus*) and three shrews (*S. murinus*) captured in flooded rice, and samples from one shrew (*S. etruscus*) and one tenrec (*M. brevicaudata*) captured in the secondary forest were also in this clade (Fig. 5). The remaining two clades, *Leptospira* species *b* (*n* = 2) and *c* (*n* = 2), were similar to *Leptospira* species *a* in that they were found in *Rousettus madagascariensis* trapped in agricultural habitats (Fig. 5).

## DISCUSSION

Land-use change alters small mammal communities, which have cascading effects on parasite communities and zoonotic risk (70). Through our multi-year, multi-location effort, we found that *Leptospira* prevalence and which bacterial species are present vary across land-use types corresponding to shifts in host communities. We found that the species composition of bats and terrestrial small mammals varied between habitat types, with notable shifts between forested habitats, agricultural land-use types, and the village homes. *Leptospira* infecting these animals also changed from predominantly cosmopolitan lineages (i.e., *L. interrogans* and *L. kirschneri*) in disturbed habitats to endemic lineages (*L. borgpetersenii* and *L. mayottensis* [53, 57, 71]) in more intact, forested habitats. This resulted in community-level infection prevalence varying with species composition between habitats and *Leptospira* shifting from endemic-associated lineages in forested habitats to cosmopolitan lineages in agricultural fields and village homes, where invasive Muridae rodents were abundant. Overall, *Leptospira* infection prevalence was much higher in bats (37.7%) than in terrestrial small mammals (13.8%).

The changes in species composition between habitat types were primarily due to changes in the relative abundance of a small number of species and, to a lesser extent, species diversity. While the study site and our samples exhibited high species richness (28 species of terrestrial mammal and 12 species of bats were captured), most individuals were of just a few species: *Rattus rattus*, *Microgale brevicaudata*, and *Mus musculus* among the terrestrial animals and *Myotis goudoti* and *Rousettus madagascariensis* among bats. In terrestrial small mammals, introduced Muridae rodents were the primary species captured outside of the national park, and many native species were limited to the forest interior. These differences in animal captures resulted in higher terrestrial animal density and lower species richness in the agricultural matrix than in forested habitats, except for richness in flooded rice fields. As hypothesized, and as is typical of many insular terrestrial small mammal systems (32), we found that invasive species replaced natives, particularly in disturbed land-use types.

Bat communities transitioned from being *M. goudoti-*dominated in forested and brushy regrowth habitat types to *R. madagascariensis-*dominated in agricultural land-use types, with agroforest having roughly equal proportions of these two species. The differences in bat communities between forested and agricultural land-use types were primarily due to the abundance of these two species, not metrics of diversity. The lack of a strong relationship between habitat and species composition may reflect that bats, with their greater dispersal capabilities via flight and associated home ranges, were less restricted to the habitat in which they were captured, since trapping location was likely more reflective of where animals feed than where they roost (72, 73). This may also be in part due to some species of bats benefiting from human-modified habitats such as villages, agricultural fields, and agroforests, because these sites can be used for their roosting and foraging activities (74). Therefore, shifts in animal density and which terrestrial small mammal and bat species were most common transitioned between forested and anthropogenic land-use types to favor non-native small mammals and fruit bats (*R. madagascariensis*).

The most abundant terrestrial mammal host species in the anthropogenic land-use types tended to be more highly infected. The synanthropic *Mus musculus* was the primary host of *Leptospira* in this region (36.8% overall prevalence), and the majority (54%) of mice captured in flooded rice fields were infected. Nevertheless, *R. rattus* remains an important host due to its higher abundance — they accounted for 43.7% of animals captured — despite lower infection prevalence (13.9%). In absolute terms, more individual *R. rattus* were infected than any other species in our study. This result sharply contrasts with previous findings from the capital city of Antananarivo, where *Mus* were highly abundant but rarely infected, compared to *Rattus norvegicus* (56). Interestingly, the infection prevalence in the second most abundant terrestrial mammal species, *M. brevicaudata*, was quite low (6.0%), and more broadly, prevalence was lower in native species (9.5% ± 22.2%) than in non-native species (18.7% ±11.6%).

Terrestrial small mammals captured in flooded rice fields were the most likely to be infected, followed by those captured in the semi-intact and villages. The semi-permanent water in rice paddies offers optimum conditions for survival and transmission of this primarily water-borne zoonotic pathogen (75–77). However, the dense vegetation under the forest canopy and the largely year-round rain also maintain humidity on the soil surface in forest habitats, which also favors bacterial survival (19). Rice paddies are likely a source of infection for animals and Malagasy rice farmers.

Interannual and seasonal differences were stronger predictors of infection prevalence than richness or density for both terrestrial and volant animals. Prevalence in terrestrial mammals was higher in the earlier years of the study, which corresponds to the animals that were also included in a previous analysis (51). The reason for this large shift is unknown, and because bats were not captured during the earlier phase of the study, we cannot know whether a similar drop in prevalence occurred in bats. The warm, wet season, which is most favorable for *Leptospira,* corresponded to slightly higher infection prevalence in terrestrial mammals, but interestingly, the opposite trend was observed in bats. However, this unexpected, large effect of bats having a higher probability of infection in the hot, dry season may simply reflect that this was the only season when animals were captured deep in the park interior, where prevalence was highest. Furthermore, some of the regional variability in habitat type was captured in the year and seasonal model terms since trapping locations covaried with season (bats) and year (terrestrial small mammals).

*Leptospira* lineages infecting small mammals also shifted between land-use types from endemic lineages (*L. borgpetersenii* and *L. mayottensis* [53, 57, 71]) in more intact, forested habitats to predominantly cosmopolitan lineages (*L. interrogans* and *L. kirschneri*) in disturbed habitats. This change in *Leptospira* lineages paralleled the changes in host species composition as the endemic *Leptospira* lineages only infected native small mammals and bats, while the cosmopolitan lineages infected both native and non-native species. The cosmopolitan clades consisted of *L. interrogans*, *L. kirschneri,* and closely related *L*. sp. *b* and *L*. sp. *c*, and endemic clades consisted of *L. borgpetersenii*, *L. mayottensis*, and the closely related *L*. sp. *a*. Cosmopolitan *Leptospira* were primarily found in non-native terrestrial mammals, captured in the more disturbed habitat types, but were also found in non-native terrestrial mammals captured in the forested habitat types. Some of these cosmopolitan *Leptospira* lineages are known to be zoonotic. For example, we found *L. kirschneri*, haplotype (*secY^3^_22_*), which has been previously found in *Hemicentetes semispinosus*, a native tenrec species trapped in the humid forest of central-east Madagascar (64), in mice from Portugal (78), and in people from Mayotte Island (79). The *L. interrogans*, closely related to the *secY^3^_13_* haplotype, was found in non-native *R. rattus,* along with two *M. musculus* and an endemic species of tenrec, *Setifer setosus*. This haplotype was also found in dogs in nearby villages (62), *R. norvegicus* in urban areas of Guangzhou, China (80), and Kuwait, and *R. tanezumi* in Thailand (81).

All the sequences obtained from animals captured inside the national park corresponded to the endemic clades of *Leptospira*. However, we did not obtain sequences for half of the positive specimens from inside the park, where infection prevalence was quite high (45.8% ± 16.2%). While this limits our ability to fully assess whether the shift in host assemblies between forested and non-forested habitats coincides with a shift in *Leptospira* lineages, we do find some evidence that this shift occurred. The forest-associated *Myotis goudoti* and *Miniopterus* spp. were mostly infected with *L. borgpetersenii* closely related to haplotypes *secY^3^_48_* and *secY^3^_61_*. Alignments of *L. borgpetersenii secY^3^_48_* identified in bats were similar to sequences identified from endemic small mammals, including *Eliurus minor*, *Microgale principula*, and *M. majori* captured in other areas of Madagascar (64). *Leptospira mayottensis* was only found in *E. grandidieri,* a native rodent species restricted to elevational ranges between 1,250 and 1,875 m on the Marojejy Massif (82). The observed haplotype *secY^3^_56_* of *Leptospira mayottensis* was previously reported in spiny tenrecs, *Tenrec ecaudatus* (introduced to neighboring islands in the western Indian Ocean, including Mayotte), and shrew tenrecs, *Microgale thomasi* (55, 71). The restricted geographical distribution of the native host limits the dispersion of their agent pathogens, causing strong host-parasite specificity and co-radiation processes that may lead to endemism of these microorganisms (55, 64). This is the second report of *L. mayottensis* in native Malagasy rodents (Nesomyinae), which have previously been found to have mixed infections with *L. interrogans* and/or *L. borgpetersenii* (52).

Similar to previous reports of *Leptospira* in Malagasy bats, we found considerable diversity, including lineages distinct from other *Leptospira* species and clades (53, 57). The bats infected with distinct lineages of *Leptospira* most closely related to *L. kirschneri* were captured in more disturbed areas. Despite *L*. sp. *a* being most closely related to *L. mayottensis,* which was only found in terrestrial mammals in the park, it was found in *Rousettus madagascariensis* in agriculture and agroforestry. *Leptospira* sp. *b* was only found in bats (*n* = 2) captured in the flooded rice, where *L. kirschneri*, its most closely related species, was most commonly found. However, *L*. sp. *c* may be a *Rousettus-*specific strain of *Leptospira* since it was closely related to samples obtained from *R. leschenaultii* in Vietnam (83). Finally, *Myotis goudoti* were infected with a *L. interrogans* haplotype closely related to *secY^3^_9_*, which was distinct from rat-associated *L. interrogans* and did not have any clear habitat-type associations. Conclusions regarding the host specificity of these bat-associated *Leptospira* are limited because we were unable to obtain sequences for most *R. madagascariensis* samples. Next steps for describing *Leptospira* in this region are to isolate *Leptospira* from these animal hosts so they can be included in a serologic microscopic agglutination test (MAT). With a MAT test, human and animal exposures can be used to assess which animals are the hosts of zoonotic *Leptospira*, quantify the public health impacts of leptospirosis, and identify serovar-matched bacteriocin vaccines (84).

In conclusion, our study provides a new example of how the conversion of forests into agricultural fields and other anthropogenic habitats increases the prevalence of infection with potentially zoonotic organisms (85, 86). In this case, the primary mechanism involved seems to be that invasive rats and mice are the dominant terrestrial small mammal species in anthropogenic habitats. These rodents tend to have higher infection prevalence and be infected with potentially zoonotic lineages of *Leptospira*, and thus their occurrence corresponds to elevated landscape-level risk (6, 7). At our study site, these introduced rodents host globally distributed haplotypes of *L. interrogans* and *L. kirschneri*, thus suggesting that these cosmopolitan *Leptospira* were introduced to Madagascar alongside their synanthropic hosts and have altered the parasite community in the agricultural habitats where they are highly abundant.

Notably, we found little to no effect of land-use change on *Leptospira* prevalence and lineages in bats. This is likely due to differences in the ecology of bats, which are highly mobile, have large home ranges, and were captured where they fed instead of where they roost. Anthropogenic change may well be changing landscape-level infection prevalence of *Leptospira* in bats, but at a larger spatial scale than encompassed in our study. Altogether, our work demonstrates that converting forests to agricultural fields results in the replacement of native species with invasive species and their parasites when investigated at a spatial scale relevant to the host community.

## MATERIALS AND METHODS

### Sample collection

Data collection was approved by Institutional Animal Care and Use Committee at Duke University (protocol number A002-17-01 2017-2019, A262-19-12 2019-2021), and by Malagasy authorities (No. 289/17, 146/18, 280/19, 57/20, 191/20, 307/21, 357/21—MEEF/SG/DGF/DSAP/SCB). Samples from small mammals were collected between 2017 and 2021 inside Marojejy National Park and around three adjacent villages: Mandena (−14.477049, 49.8147), Manantenina (−14.497213, 49.821347), and Sarahandrano (−14.607567, 49.647759). The protected area is located in the SAVA region of northeastern Madagascar and has an area of 60,150 ha and an elevational range from 75 to 2,133 m.

**Fig 6:**
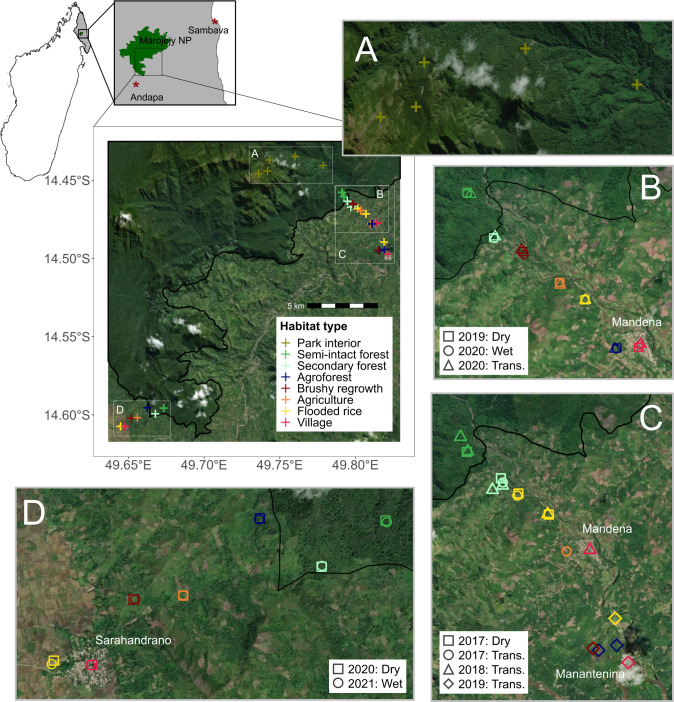
Map of the sampling sites in (**A**) Marojejy National Park interior and in the three locations (**B**) Mandena, (**C**) Mandena & Manantenina, and (**D**) Sarahandrano where animals were trapped in different land use types and during three distinct seasons: warm wet period (March–May), cool wet period (June–September), and hot dry period (October–December). Maps were made using the sf (88, 89) and basemaps (90) packages. The country and regional insets were made using shapefiles available in the geodata package (91) and from Protected Planet (92). Basemaps are Esri “World Imagery.”

Animals were captured in eight different habitat types: (i) primary forest in the park’s interior, (ii) semi-intact forest situated just inside the park, (iii) secondary forest fragments located outside the park that have experienced human or natural perturbation, (iv) agroforest containing agricultural vanilla and cultivated trees, (v) brushy regrowth areas containing shrubs and bushes occurring after swidden cultivation practice, (vi) open mixed agricultural fields with less tree cover, (vii) flooded rice fields, and (viii) in homes and other village structures. Sampling was performed during three field sessions: May 2017 to July 2019 in Mandena and Manantenina; September 2019 to September 2021 in Mandena and Sarahandrano; and October to November 2021 inside Marojejy National Park (see Fig. 6 for more details). Sherman and National traps were placed at 10 m intervals in 90 × 90 m (before September 2019) or 100 × 100 m trap grids (after September 2019). Two pitfall lines of 100 m long (all dates) were installed 20–50 m distance from the trap grid, with 11 buckets (pitfall traps) of 15 L placed every 10 m along each line. Additionally, traps were placed inside people’s homes in the villages. Bats were captured using mist nets (12 × 2.6 m or 6 × 2.6 m) and harp traps (1.0 × 2.6 m) installed along trails and paths adjacent to the terrestrial mammal trap grids. Traps placed in the park interior (not semi-intact forest) were installed along an elevational transect at five sites ranging from 480 to 1,880 m encompassing the swath of the Marojejy Massif. At each of these five sites, two trap lines of 50 traps and three pitfall lines (11 buckets spaced 10 m apart with vertical drift fencing between) were installed for seven consecutive nights. For all habitats outside of the elevational transect, the number of nights of trap effort varied between habitat type and study period, ranging from 5 to 10 consecutive nights. In total, 46,506 nights of trapping occurred (6,941 for pitfall buckets and 39,565 for traps). Non-native and a subset of native (less than 10 for rare species) animals were euthanized and necropsied for a range of different zoonotic disease projects. Relevant to this study, kidney tissues were stored in 70% ethanol and transported to the laboratory for molecular analyses, where they were stored at −20°C. The taxonomy of captured small mammals follows Soarimalala and Goodman (2011) (87).

### Tissue processing and *Leptospira* screening

Kidney tissues were rehydrated overnight in 1.5 mL of autoclaved milli-Q water to remove ethanol, then 20 mg of kidney tissue was finely cut with a sterile scalpel and lysed at 56°C for 3–6 h in 180 µL ATL buffer (QIAGEN, Valencia, California, USA) supplemented with 20 µL proteinase K (IndiSpin QIAcube HT Pathogen Kit 480). Total nucleic acids were extracted using a QIACUBE robot and the Cador Pathogen kit (Qiagen, Valencia, California, USA) per the manufacturer’s instructions with a 200 µL elution volume. Reverse transcription was performed on 10 µL of nucleic acids using the ProtoScript II Reverse Transcriptase and Random Primer 6 (New England BioLabs, Ipswich, Massachusetts, USA) under the following thermal conditions: 70°C for 5 min (denaturation 1), 4°C for 2 min, 25°C for 10 min (denaturation 2), 42°C for 50 min (annealing), and 65°C for 20 min (extension). Reverse transcription was used to screen both RNA (viral) and DNA pathogens with the same cDNA preparation.

The presence of *Leptospira* in the samples was tested using a previously published probe-specific real-time PCR targeting bacterial 16S (93), and samples with a cycle threshold (Ct) value < 40 were considered positive. Five microliters of cDNA was amplified in a reaction mixture composed of 10 µL of Quanti Nova Probe PCR (Qiagen), 0.5 µL (10 µM) of each primer (reverse and forward), 0.4 µL (10 µM) of the probe, and 3.6 µL of RNAse-free water. Thermal conditions consisted of an initial denaturation step (95°C for 2 min) followed by 45 cycles of annealing (95°C for 5 s) and extension (60°C for 5 s). For all positive samples (Ct value < 40)*, Leptospira* were characterized at the species level using the *secY* gene, a housekeeping gene commonly used for species identification of pathogenic *Leptospira* infecting small mammals (64). For this, 2 µL of cDNA from each RT-PCR-positive sample was used as PCR template in a reaction mixture composed of 12.5 µL of GoTaq G2 Hot Start Green Master Mix (Promega, Madison, Wisconsin, USA), 0.5 µL (10 µM) of each primer, and 9.5 µL of RNAse-free water. PCR conditions were as follows: a first GoTaq activation step (95°C for 2 min), then 45 cycles of denaturation (94°C for 30 s), annealing (58°C for 30 s), and extension (72°C for 1 min), then a final extension step (72°C for 5 min). When we failed to obtain sequences with standard *secY* primers (94), further analysis using alternative degenerate (64) and *secYinner* primers (95) was performed. Sanger sequencing was carried out on both strands (Genoscreen, Lille, France), and the obtained chromatograms were manually aligned and edited using Geneious software v.9.1.8.

### Statistical analysis

All statistical analysis was performed in R software v.4.4.1 (96). We visualized patterns in small mammal communities in association with land use type by using NMDS analysis. Next, we used a PERMANOVA to test whether the community composition of small mammals and a PERMDIST to test whether community variability (dispersion) differed across habitat types. For each habitat type, we calculated species richness (the number of species of small mammals or bats), Shannon diversity (accounts for the abundance and evenness of the species present), and evenness using the diversity function in the “vegan” package (97). An ANOVA followed by a Tukey’s post hoc test for pairwise comparisons was used to compare species richness and diversity between habitat types.

To investigate the effect of environmental variables and species composition on *Leptospira* prevalence, we used binomial models of the number of infected animals given the number tested in each trapping period (i.e., the 5- to 10-day period when traps were installed in each habitat type in each season). In the models of the terrestrial mammal infections, the environmental variables were the type of habitat (park interior, semi-intact forest, secondary forest, agroforest, brushy regrowth, agriculture, flooded rice, and village), season (warm wet, cool wet, and hot dry), year (2017, 2018, 2019, 2020, and 2021), and locality (Mandena, Manantenina, Sarahandrano, and park interior). The species composition variables included Shannon diversity, species richness, evenness, animal density (number of small mammals trapped per night of trap effort), and the proportion of captured animals that were native species. In the models of bat infections, the environmental variables included the same habitat types, but year (2019 and 2021), season (hot dry and cool wet), and locality (Mandena, Sarahandrano, and park interior) were modified to include just the periods in which bats were captured. The species composition variables in the models of bat infections were Shannon diversity, richness, evenness, and the number of animals captured at each location. Due to issues with multicollinearity and singularity, Shannon diversity and locality were dropped from both sets of models.

To identify which factors best predict infection with *Leptospira*, all combinations of predictors were performed using the “dredge” function with the “MuMIn” package (98). The best model subset, containing models with differences less than two of the corrected Akaike Information Criterion (AIC), was then used to find variable importance (sum AIC weight for all models in the best subset containing the variable). The full model-averaged coefficients were used to describe the effect of each variable on the odds of detecting animals infected with *Leptospira* during each trapping period.

### Phylogenetic analysis

Phylogenetic analysis was performed with all *secY* sequences obtained and 15 reference sequences that were aligned with Seaview software v.5. The phylogenetic tree was inferred using ML with default settings, including a General Time Reversible model for base pair substitutions and 1000 bootstrap replicates, and then edited with FigTree software v.1.4.4. Allelic numbers for the *secY* gene were assigned using the Public PubMLST database (scheme#3). *Leptospira* species assignment was carried out using the *Leptospira* isolates database available on PUBMLST, and 783 fully or partly genotyped strains were used with *secY*^3^ allele determination.

## Data Availability

Sequence data were deposited in GenBank under the following accession numbers: MT811060-MT811094, OR189380-OR189405, OQ946505-OQ946538, and PP895334-PP895362.

## AFTER EPUB

*[This article was published on 30 January 2026 with a typographical error in the Importance. The error was corrected in the current version, posted on 2 February 2026.]*

## SUPPLEMENTAL MATERIAL

Supplemental material aem.02061-25-s0001.docx Figures S1 to S3; Tables S1 to S4.

Fig. S4 aem.02061-25-s0002.html Interactive Sankey diagram showing the patterns of land use gradients, small mammal host species, and *Leptospira* species.

ASM does not own the copyrights to Supplemental Material that may be linked to, or accessed through, an article. The authors have granted ASM a non-exclusive, world-wide license to publish the Supplemental Material files. Please contact the corresponding author directly for reuse.

## References

[1] Carlson CJ, Brookson CB, Becker DJ, Cummings CA, Gibb R, Halliday FW, Heckley AM, Huang ZYX, Lavelle T, Robertson H, Vicente-Santos A, Weets CM, Poisot T. 2025. Pathogens and planetary change. Nat Rev Biodivers 1:32–49. doi:10.1038/s44358-024-00005-w PMC7618822 41789152

[2] Faust CL, McCallum HI, Bloomfield LSP, Gottdenker NL, Gillespie TR, Torney CJ, Dobson AP, Plowright RK. 2018. Pathogen spillover during land conversion. Ecol Lett 21:471–483. doi:10.1111/ele.12904 29466832

[3] Lambin EF, Tran A, Vanwambeke SO, Linard C, Soti V. 2010. Pathogenic landscapes: interactions between land, people, disease vectors, and their animal hosts. Int J Health Geogr 9:54. doi:10.1186/1476-072X-9-54 20979609 PMC2984574

[4] Johnson PTJ, Preston DL, Hoverman JT, Richgels KLD. 2013. Biodiversity decreases disease through predictable changes in host community competence. Nature 494:230–233. doi:10.1038/nature11883 23407539

[5] Glidden CK, Nova N, Kain MP, Lagerstrom KM, Skinner EB, Mandle L, Sokolow SH, Plowright RK, Dirzo R, De Leo GA, Mordecai EA. 2021. Human-mediated impacts on biodiversity and the consequences for zoonotic disease spillover. Curr Biol 31:R1342–R1361. doi:10.1016/j.cub.2021.08.070 34637744 PMC9255562

[6] McFarlane R, Sleigh A, McMichael T. 2012. Synanthropy of wild mammals as a determinant of emerging infectious diseases in the Asian-Australasian region. Ecohealth 9:24–35. doi:10.1007/s10393-012-0763-9 22526750 PMC7088064

[7] Gibb R, Redding DW, Chin KQ, Donnelly CA, Blackburn TM, Newbold T, Jones KE. 2020. Zoonotic host diversity increases in human-dominated ecosystems. Nature 584:398–402. doi:10.1038/s41586-020-2562-8 32759999

[8] Becker DJ, Streicker DG, Altizer S. 2018. Using host species traits to understand the consequences of resource provisioning for host-parasite interactions. J Anim Ecol 87:511–525. doi:10.1111/1365-2656.12765 29023699 PMC5836909

[9] Johnson PTJ, Rohr JR, Hoverman JT, Kellermanns E, Bowerman J, Lunde KB. 2012. Living fast and dying of infection: host life history drives interspecific variation in infection and disease risk. Ecol Lett 15:235–242. doi:10.1111/j.1461-0248.2011.01730.x 22221837

[10] Young HS, Dirzo R, Helgen KM, McCauley DJ, Billeter SA, Kosoy MY, Osikowicz LM, Salkeld DJ, Young TP, Dittmar K. 2014. Declines in large wildlife increase landscape-level prevalence of rodent-borne disease in Africa. Proc Natl Acad Sci USA 111:7036–7041. doi:10.1073/pnas.1404958111 24778215 PMC4024866

[11] Borremans B, Faust C, Manlove KR, Sokolow SH, Lloyd-Smith JO. 2019. Cross-species pathogen spillover across ecosystem boundaries: mechanisms and theory. Phil Trans R Soc B 374:20180344. doi:10.1098/rstb.2018.0344 31401953 PMC6711298

[12] da Cunha CEP, Felix SR, Neto ACPS, Campello-Felix A, Kremer FS, Monte LG, Amaral MG, de Oliveira Nobre M, da Silva ÉF, Hartleben CP, McBride AJA, Dellagostin OA. 2016. Infection with Leptospira kirschneri serovar mozdok: first report from the southern hemisphere. Am J Trop Med Hyg 94:519–521. doi:10.4269/ajtmh.15-0505 26755566 PMC4775884

[13] Olival KJ, Hosseini PR, Zambrana-Torrelio C, Ross N, Bogich TL, Daszak P. 2017. Host and viral traits predict zoonotic spillover from mammals. Nature 546:646–650. doi:10.1038/nature22975 28636590 PMC5570460

[14] Patz JA, Daszak P, Tabor GM, Aguirre AA, Pearl M, Epstein J, Wolfe ND, Kilpatrick AM, Foufopoulos J, Molyneux D, Bradley DJ. 2004. Unhealthy landscapes: policy recommendations on land use change and infectious disease emergence. Environ Health Perspect 112:1092–1098. doi:10.1289/ehp.6877 15238283 PMC1247383

[15] LoGiudice K, Ostfeld RS, Schmidt KA, Keesing F. 2003. The ecology of infectious disease: effects of host diversity and community composition on Lyme disease risk. Proc Natl Acad Sci USA 100:567–571. doi:10.1073/pnas.0233733100 12525705 PMC141036

[16] Alderson J, Quastel M, Wilson E, Bellamy D. 2020. Factors influencing the re-emergence of plague in Madagascar. Emerg Top Life Sci 4:411–421. doi:10.1042/ETLS20200334 33258957 PMC7733672

[17] Field H, Young P, Yob JM, Mills J, Hall L, Mackenzie J. 2001. The natural history of Hendra and Nipah viruses. Microbes Infect 3:307–314. doi:10.1016/s1286-4579(01)01384-3 11334748

[18] Glidden CK, Murran AR, Silva RA, Castellanos AA, Han BA, Mordecai EA. 2023. Phylogenetic and biogeographical traits predict unrecognized hosts of zoonotic leishmaniasis. PLoS Negl Trop Dis 17:e0010879. doi:10.1371/journal.pntd.0010879 37256857 PMC10231829

[19] Bierque E, Thibeaux R, Girault D, Soupé-Gilbert M-E, Goarant C. 2020. A systematic review of Leptospira in water and soil environments. PLoS One 15:e0227055. doi:10.1371/journal.pone.0227055 31986154 PMC6984726

[20] Manlove K, Wilber M, White L, Bastille‐Rousseau G, Yang A, Gilbertson MLJ, Craft ME, Cross PC, Wittemyer G, Pepin KM. 2022. Defining an epidemiological landscape that connects movement ecology to pathogen transmission and pace‐of‐life. Ecol Lett 25:1760–1782. doi:10.1111/ele.14032 35791088

[21] Salzer JS, Pinto CM, Grippi DC, Williams-Newkirk AJ, Peterhans JK, Rwego IB, Carroll DS, Gillespie TR. 2016. Impact of anthropogenic disturbance on native and invasive trypanosomes of rodents in forested Uganda. Ecohealth 13:698–707. doi:10.1007/s10393-016-1160-6 27655649

[22] Nunn CL, Solis A, Rakotonarivo OS, Golden CD, Kramer RA. 2022. One Health research and practice on Madagascar, p 247–264. In Goodman SM (ed), The new natural history of Madagascar. Princeton University Press, Princeton, NJ, USA.

[23] Allen T, Murray KA, Zambrana-Torrelio C, Morse SS, Rondinini C, Di Marco M, Breit N, Olival KJ, Daszak P. 2017. Global hotspots and correlates of emerging zoonotic diseases. Nat Commun 8:1124. doi:10.1038/s41467-017-00923-8 29066781 PMC5654761

[24] Midgley A, Golden CD, Bouley T, Shumake-Guillemot J, Ebi KL. 2018. Climate change and health diagnostic: risks and opportunities for climate-smart health and nutrition investment. Available from: http://documents.worldbank.org/curated/en/936661516004441146

[25] Goodman SM, Raselimanana AP, Tahinarivony JA. 2023. Description of the Parc National de Marojejy, Madagascar, and the 2021 biological inventory of the massif. In A floral and faunal inventory of the Parc National de Marojejy: altitudinal gradient and temporal variation. Edited by S. M. Goodman and M. J. Raherilalao. Malagasy Nature 17:5–31.

[26] Han BA, Schmidt JP, Bowden SE, Drake JM. 2015. Rodent reservoirs of future zoonotic diseases. Proc Natl Acad Sci USA 112:7039–7044. doi:10.1073/pnas.1501598112 26038558 PMC4460448

[27] Raharinosy V, Olive M-M, Andriamiarimanana FM, Andriamandimby SF, Ravalohery J-P, Andriamamonjy S, Filippone C, Rakoto DAD, Telfer S, Heraud J-M. 2018. Geographical distribution and relative risk of Anjozorobe virus (Thailand orthohantavirus) infection in black rats (Rattus rattus) in Madagascar. Virol J 15:83. doi:10.1186/s12985-018-0992-9 29743115 PMC5944027

[28] Reynes J-M, Razafindralambo NK, Lacoste V, Olive M-M, Barivelo TA, Soarimalala V, Heraud J-M, Lavergne A. 2014. Anjozorobe hantavirus, a new genetic variant of Thailand virus detected in rodents from Madagascar. Vector Borne Zoonotic Dis 14:212–219. doi:10.1089/vbz.2013.1359 24575755 PMC3952587

[29] Lebarbenchon C, Ramasindrazana B, Joffrin L, Bos S, Lagadec E, Le Minter G, Gomard Y, Tortosa P, Wilkinson DA, Goodman SM, Mavingui P. 2017. Astroviruses in bats, Madagascar. Emerg Microbes Infect 6:e58. doi:10.1038/emi.2017.47 28634357 PMC5520320

[30] Carcauzon V, Herrera JP, Kaufman K, Baudino F, Wickenkamp N, Randriamoria TM, Soarimalala V, Goodman SM, Nunn CL, Lebarbenchon C, Tortosa P. 2024. Astroviruses in terrestrial Malagasy mammals. PLoS Negl Trop Dis 18:e0012263. doi:10.1371/journal.pntd.0012263 38875307 PMC11262628

[31] Wilkinson DA, Mélade J, Dietrich M, Ramasindrazana B, Soarimalala V, Lagadec E, le Minter G, Tortosa P, Heraud J-M, de Lamballerie X, Goodman SM, Dellagi K, Pascalis H. 2014. Highly diverse morbillivirus-related paramyxoviruses in wild fauna of the southwestern Indian Ocean Islands: evidence of exchange between introduced and endemic small mammals. J Virol 88:8268–8277. doi:10.1128/JVI.01211-14 24829336 PMC4135957

[32] Duplantier J-M, Duchemin J-B, Ramandimbilahatra L, Goodman SM. 2003. Human diseases and introduced small mammals, p 158–161. *In* Goodman SM, Benstead JP (ed), The natural history of Madagascar. University of Chicago.

[33] Rahalison L. 2003. Susceptibility to plague of the rodents in Antananarivo, Madagascar, p 439–442. In Skurnik M, Bengoechea JA, Granfors K (ed), The genus Yersinia: entering the functional genomic era. Springer US, Boston, MA. 10.1007/0-306-48416-1_87 12756805

[34] Bublitz DC, Wright PC, Bodager JR, Rasambainarivo FT, Bliska JB, Gillespie TR. 2014. Epidemiology of pathogenic enterobacteria in humans, livestock, and peridomestic rodents in rural Madagascar. PLoS One 9:e101456. doi:10.1371/journal.pone.0101456 24983990 PMC4077799

[35] Collard J-M, Andrianonimiadana L, Habib A, Rakotondrainipiana M, Andriantsalama P, Randriamparany R, Rabenandrasana MAN, Weill F-X, Sauvonnet N, Randremanana RV, Guillemot V, Vonaesch P, Sansonetti PJ. 2022. High prevalence of small intestine bacteria overgrowth and asymptomatic carriage of enteric pathogens in stunted children in Antananarivo, Madagascar. PLoS Negl Trop Dis 16:e0009849. doi:10.1371/journal.pntd.0009849 35533199 PMC9119516

[36] Rahelinirina S, Léon A, Harstskeerl RA, Sertour N, Ahmed A, Raharimanana C, Ferquel E, Garnier M, Chartier L, Duplantier J-M, Rahalison L, Cornet M. 2010. First isolation and direct evidence for the existence of large small-mammal reservoirs of Leptospira sp. in Madagascar. PLoS One 5:e14111. doi:10.1371/journal.pone.0014111 21124843 PMC2991340

[37] Pagès F, Kuli B, Moiton M-P, Goarant C, Jaffar-Bandjee M-C. 2015. Leptospirosis after a stay in Madagascar. J Travel Med 22:136–139. doi:10.1111/jtm.12163 25319525

[38] Vieilledent G, Grinand C, Rakotomalala FA, Ranaivosoa R, Rakotoarijaona J-R, Allnutt TF, Achard F. 2018. Combining global tree cover loss data with historical national forest cover maps to look at six decades of deforestation and forest fragmentation in Madagascar. Biol Conserv 222:189–197. doi:10.1016/j.biocon.2018.04.008

[39] Harvey CA, Rakotobe ZL, Rao NS, Dave R, Razafimahatratra H, Rabarijohn RH, Rajaofara H, MacKinnon JL. 2014. Extreme vulnerability of smallholder farmers to agricultural risks and climate change in Madagascar. Phil Trans R Soc B 369:20130089. doi:10.1098/rstb.2013.0089 24535397 PMC3928894

[40] Sarfo Y, Musshoff O, Weber R, Danne M. 2021. Farmers’ willingness to pay for digital and conventional credit: insight from a discrete choice experiment in Madagascar. PLoS One 16:e0257909. doi:10.1371/journal.pone.0257909 34767559 PMC8589200

[41] Hume DW. 2006. Swidden agriculture and conservation in eastern Madagascar: stakeholder perspectives and cultural belief systems. Conserv Soc 4:287–303.

[42] Andriatsitohaina RNN, Laby P, Llopis JC, Martin DA. 2024. Agroforestry in Madagascar: past, present, and future. Agrofor Syst 98:1659–1680. doi:10.1007/s10457-024-00975-y 39086741 PMC11286679

[43] Laney R, Turner BL. 2015. The persistence of self-provisioning among smallholder farmers in northeast Madagascar. Hum Ecol 43:811–826. doi:10.1007/s10745-015-9791-8 PMC4673095 26691538

[44] Mariel J, Penot E, Labeyrie V, Herimandimby H, Danthu P. 2023. From shifting rice cultivation (tavy) to agroforestry systems: a century of changing land use on the east coast of Madagascar. Agroforest Syst 97:415–431. doi:10.1007/s10457-022-00761-8

[45] Andriamparany JN, Hänke H, Schlecht E. 2021. Food security and food quality among vanilla farmers in Madagascar: the role of contract farming and livestock keeping. Food Sec 13:981–1012. doi:10.1007/s12571-021-01153-z

[46] Jégourel Y. 2021. La vanille malgache: entre valorisation des avantages comparatifs et nécessaire diversification économique. Policy Briefs. https://ideas.repec.org//p/ocp/ppaper/pb45-21.html.

[47] Celio E, Andriatsitohaina RNN, Llopis JC, Gret-Regamey A. 2023. Assessing farmers’ income vulnerability to vanilla and clove export economies in northeastern Madagascar using land-use change modelling. J Land Use Sci 18:55–83. doi:10.1080/1747423X.2023.2168778

[48] Stoop WA, Uphoff N, Kassam A. 2002. A review of agricultural research issues raised by the system of rice intensification (SRI) from Madagascar: opportunities for improving farming systems for resource-poor farmers. Agric Syst 71:249–274. doi:10.1016/S0308-521X(01)00070-1

[49] Wurz A, Grass I, Lees DC, Rakotomalala AANA, Sáfián S, Martin DA, Osen K, Loos J, Benasoavina E, Alexis T, Tscharntke T. 2022. Land‐use change differentially affects endemic, forest and open‐land butterflies in Madagascar. Insect Conserv Diversity 15:606–620. doi:10.1111/icad.12580

[50] Rahelinirina S, Bourhy P, Andriamiaramanana F, Garin B, Rajerison M. 2019. High prevalence of Leptospira spp. in rodents in an urban setting in Madagascar. Am J Trop Med Hyg 100:1079–1081. doi:10.4269/ajtmh.18-0642 30915950 PMC6493950

[51] Herrera JP, Wickenkamp NR, Turpin M, Baudino F, Tortosa P, Goodman SM, Soarimalala V, Ranaivoson TN, Nunn CL. 2020. Effects of land use, habitat characteristics, and small mammal community composition on Leptospira prevalence in northeast Madagascar. PLoS Negl Trop Dis 14:e0008946. doi:10.1371/journal.pntd.0008946 33382723 PMC7774828

[52] Moseley M, Rahelinirina S, Rajerison M, Garin B, Piertney S, Telfer S. 2018. Mixed Leptospira infections in a diverse reservoir host community, Madagascar, 2013-2015. Emerg Infect Dis 24:1138–1140. doi:10.3201/eid2406.180035 29774844 PMC6004868

[53] Gomard Y, Dietrich M, Wieseke N, Ramasindrazana B, Lagadec E, Goodman SM, Dellagi K, Tortosa P. 2016. Malagasy bats shelter a considerable genetic diversity of pathogenic Leptospira suggesting notable host-specificity patterns. FEMS Microbiol Ecol 92:fiw037. doi:10.1093/femsec/fiw037 26902801

[54] Desvars A, Michault A, Bourhy P. 2013. Leptospirosis in the Western Indian Ocean Islands: what is known so far? Vet Res 44:80. doi:10.1186/1297-9716-44-80 24016311 PMC3852700

[55] Gomard Y, Goodman SM, Soarimalala V, Turpin M, Lenclume G, Ah-Vane M, Golden CD, Tortosa P. 2022. Co-radiation of Leptospira and Tenrecidae (Afrotheria) on Madagascar. Trop Med Infect Dis 7:193. doi:10.3390/tropicalmed7080193 36006285 PMC9415048

[56] Rahelinirina S, Moseley MH, Allan KJ, Ramanohizakandrainy E, Ravaoarinoro S, Rajerison M, Rakotoharinome V, Telfer S. 2019. Leptospira in livestock in Madagascar: uncultured strains, mixed infections and small mammal-livestock transmission highlight challenges in controlling and diagnosing leptospirosis in the developing world. Parasitology 146:1707–1713. doi:10.1017/S0031182019001252 31554531 PMC6935375

[57] Dietrich M, Gomard Y, Lagadec E, Ramasindrazana B, Le Minter G, Guernier V, Benlali A, Rocamora G, Markotter W, Goodman SM, Dellagi K, Tortosa P. 2018. Biogeography of Leptospira in wild animal communities inhabiting the insular ecosystem of the Western Indian Ocean Islands and neighboring Africa. Emerg Microbes Infect 7:1–12. doi:10.1038/s41426-018-0059-4 29615623 PMC5883017

[58] Dubrulle J, Kauffman K, Soarimalala V, Randriamoria T, Goodman SM, Herrera J, Nunn C, Tortosa P. 2025. Effect of land‐use on Hantavirus infection among introduced and endemic small mammals of Madagascar. Ecol Evol 15:e70914. doi:10.1002/ece3.70914 40196405 PMC11975053

[59] Cordonin C, Turpin M, Bringart M, Bascands J-L, Flores O, Dellagi K, Mavingui P, Roche M, Tortosa P. 2020. Pathogenic Leptospira and their animal reservoirs: testing host specificity through experimental infection. Sci Rep 10:7239. doi:10.1038/s41598-020-64172-4 32350316 PMC7190861

[60] Levett PN. 2001. Leptospirosis. Clin Microbiol Rev 14:296–326. doi:10.1128/CMR.14.2.296-326.2001 11292640 PMC88975

[61] Costa F, Hagan JE, Calcagno J, Kane M, Torgerson P, Martinez-Silveira MS, Stein C, Abela-Ridder B, Ko AI. 2015. Global morbidity and mortality of leptospirosis: a systematic review. PLoS Negl Trop Dis 9:e0003898. doi:10.1371/journal.pntd.0003898 26379143 PMC4574773

[62] Kauffman K. 2024. Assessing the cross-species effects of dog Leptospira vaccinations in rural northeastern Madagascar. SSRN Scholarly Paper. Available from: 10.2139/ssrn.4719184

[63] Schafbauer T, Dreyfus A, Hogan B, Rakotozandrindrainy R, Poppert S, Straubinger RK. 2014. Seroprevalence of Leptospira spp. infection in cattle from central and northern Madagascar. IJERPH 16:2014. doi:10.3390/ijerph16112014 PMC6603958 31174244

[64] Dietrich M, Wilkinson DA, Soarimalala V, Goodman SM, Dellagi K, Tortosa P. 2014. Diversification of an emerging pathogen in a biodiversity hotspot: Leptospira in endemic small mammals of Madagascar. Mol Ecol 23:2783–2796. doi:10.1111/mec.12777 24784171

[65] Ratsitorahina M, Rahelinirina S, Michault A, Rajerison M, Rajatonirina S, Richard V. 2015. Has Madagascar lost its exceptional leptospirosis free-like status? PLoS One 10:e0122683. doi:10.1371/journal.pone.0122683 25874381 PMC4396993

[66] Rajaonarivelo JA, Desmoulin A, Maillard O, Collet L, Baudino F, Jaffar-Bandjee M-C, Blondé R, Raffray L, Tortosa P. 2023. Clinical manifestations of human leptospirosis: bacteria matter. Front Cell Infect Microbiol 13:1259599. doi:10.3389/fcimb.2023.1259599 37953799 PMC10635415

[67] Desmoulin A, Rajaonarivelo A, Maillard O, Collet L, Jaffar-Bandjee M-C, Moiton M-P, Poubeau P, Fayeulle S, François-Wattrelot C, Blondé R, Tortosa P, Raffray L. 2024. A comparative study of human leptospirosis between Mayotte and Reunion islands highlights distinct clinical and microbial features arising from distinct inter-island bacterial ecology. Am J Trop Med Hyg 111:237–245. doi:10.4269/ajtmh.23-0846 38955193 PMC11310607

[68] Cordonin C, Turpin M, Bascands J-L, Dellagi K, Mavingui P, Tortosa P, Roche M. 2019. Three Leptospira strains from western Indian Ocean wildlife show highly distinct virulence phenotypes through hamster experimental infection. Front Microbiol 10:382. doi:10.3389/fmicb.2019.00382 30915044 PMC6421516

[69] Sjoberg D. 2021. Ggsankey: Sankey, alluvial and Sankey bump plots

[70] Lafferty K, Torchin ME, Kuris A. 2010. The geography of host and parasite invasions, p 191–203. In Morand S, Krasnov BR (ed), Biogeogr. host-parasite interact. Oxford University Press.

[71] Lagadec E, Gomard Y, Le Minter G, Cordonin C, Cardinale E, Ramasindrazana B, Dietrich M, Goodman SM, Tortosa P, Dellagi K. 2016. Identification of Tenrec ecaudatus, a wild mammal introduced to Mayotte Island, as a reservoir of the newly identified human pathogenic Leptospira mayottensis. PLoS Negl Trop Dis 10:e0004933. doi:10.1371/journal.pntd.0004933 27574792 PMC5004980

[72] Park KJ. 2015. Mitigating the impacts of agriculture on biodiversity: bats and their potential role as bioindicators. Mamm Biol 80:191–204. doi:10.1016/j.mambio.2014.10.004

[73] Monck-Whipp L, Martin AE, Francis CM, Fahrig L. 2018. Farmland heterogeneity benefits bats in agricultural landscapes. Agric Ecosyst Environ 253:131–139. doi:10.1016/j.agee.2017.11.001

[74] Randrianandrianina F, Andriafidison D, Kofoky AF, Ramilijaona O, Ratrimomanarivo F, Racey PA, Jenkins RKB. 2006. Habitat use and conservation of bats in rainforest and adjacent human-modified habitats in eastern Madagascar. Acta Chiropterol 8:429–437. doi:10.3161/1733-5329(2006)8[429:HUACOB]2.0.CO;2

[75] Yapi GY, Touré M, Sarr MD, Abo N, Diabaté S. 2017. The impact of irrigated rice on the transmission of schistosomiasis and geohelminthiasis in Niakaramandougou, Côte d’Ivoire. Int J Bio Chem Sci 11:1400. doi:10.4314/ijbcs.v11i4.1

[76] Kopolrat K, Sithithaworn P, Kiatsopit N, Namsanor J, Laoprom N, Tesana S, Andrews RH, Petney TN. 2020. Influence of water irrigation schemes and seasonality on transmission dynamics of Opisthorchis viverrini in the snail intermediate host, Bithynia siamensis goniomphalos in rice paddy fields in Northeast Thailand. Am J Trop Med Hyg 103:276–286. doi:10.4269/ajtmh.19-0290 32394873 PMC7356465

[77] Chuah CJ, Tan EKH, Sermswan RW, Ziegler AD. 2017. Hydrological connectivity and Burkholderia pseudomallei prevalence in wetland environments: investigating rice-farming community’s risk of exposure to melioidosis in North-East Thailand. Environ Monit Assess 189:287. doi:10.1007/s10661-017-5988-1 28536911

[78] Ferreira AS, Ahmed A, Rocha T, Vieira ML, Paiva-Cardoso M das N, Mesquita JR, van der Linden H, Goris M, Thompson G, Hartskeerl RA, Inácio J. 2020. Genetic diversity of pathogenic leptospires from wild, domestic and captive host species in Portugal. Transbound Emerg Dis 67:852–864. doi:10.1111/tbed.13409 31677243

[79] Bourhy P, Collet L, Lernout T, Zinini F, Hartskeerl RA, van der Linden H, Thiberge JM, Diancourt L, Brisse S, Giry C, Pettinelli F, Picardeau M. 2012. Human Leptospira isolates circulating in Mayotte (Indian Ocean) have unique serological and molecular features. J Clin Microbiol 50:307–311. doi:10.1128/JCM.05931-11 22162544 PMC3264139

[80] Shao J-W, Wei Y-H, Yao X-Y, Chen H-Y, Liu H, Sun J, Chen S-Y. 2022. Pathogenic Leptospira species are widely disseminated among wild rodents in urban areas of Guangzhou, Southern China. Microorganisms 10:873. doi:10.3390/microorganisms10050873 35630318 PMC9147055

[81] Cosson J-F, Picardeau M, Mielcarek M, Tatard C, Chaval Y, Suputtamongkol Y, Buchy P, Jittapalapong S, Herbreteau V, Morand S. 2014. Epidemiology of Leptospira transmitted by rodents in southeast Asia. PLoS Negl Trop Dis 8:e2902. doi:10.1371/journal.pntd.0002902 24901706 PMC4046967

[82] Carleton M, Goodman S. 2000. Edited by SM Goodman. Rodents of the Parc National de Marojejy, Madagascar. In a floral and faunal inventory of the Parc National de Marojejy, Madagascar: with reference to elevational variation, p 231–263. Fieldiana Zoology.

[83] Radyuk EV, Breneva NV, Budaeva SE, Makenov MT, Stukolova OА, Bulanenko VP, Le LAT, Dao MN, Nguyen CV, Bui Thi NT, Luong MT, Nguyen TN, Balakhonov SV, Karan LS. 2024. Leptospira infection in bats in Vietnam. Acta Trop 257:107298. doi:10.1016/j.actatropica.2024.107298 38909726

[84] Silveira MM, Oliveira TL, Schuch RA, McBride AJA, Dellagostin OA, Hartwig DD. 2017. DNA vaccines against leptospirosis: a literature review. Vaccine 35:5559–5567. doi:10.1016/j.vaccine.2017.08.067 28882437

[85] Shah HA, Huxley P, Elmes J, Murray KA. 2019. Agricultural land-uses consistently exacerbate infectious disease risks in southeast Asia. Nat Commun 10:4299. doi:10.1038/s41467-019-12333-z 31541099 PMC6754503

[86] White RJ, Razgour O. 2020. Emerging zoonotic diseases originating in mammals: a systematic review of effects of anthropogenic land‐use change. Mamm Rev 50:336–352. doi:10.1111/mam.12201 32836691 PMC7300897

[87] Soarimalala V, Goodman SM. 2011. Les petits mammifères de Madagascar. Association Vahatra in Antananarivo.

[88] Pebesma E. 2018. Simple features for r: standardized support for spatial vector data. R J 10:439. doi:10.32614/RJ-2018-009

[89] Pebesma E, Bivand R. 2023. Spatial data science: with applications in R. Chapman and Hall/CRC, New York.

[90] Schwalb-Willmann J. 2024. basemaps: accessing spatial basemaps in R

[91] Hijmans RJ, Barbosa M, Ghosh A, Mandel A. 2024. Geodata: download geographic data

[92] Protected Areas (WDPA). 2024 Protected planet. Available from: https://www.protectedplanet.net./en/thematic-areas/wdpa

[93] Smythe LD, Smith IL, Smith GA, Dohnt MF, Symonds ML, Barnett LJ, McKay DB. 2002. A quantitative PCR (TaqMan) assay for pathogenic Leptospira spp. BMC Infect Dis 2:13. doi:10.1186/1471-2334-2-13 12100734 PMC117785

[94] Ahmed N, Devi SM, Valverde M de los A, Vijayachari P, Machang’u RS, Ellis WA, Hartskeerl RA. 2006. Multilocus sequence typing method for identification and genotypic classification of pathogenic Leptospira species. Ann Clin Microbiol Antimicrob 5:28. doi:10.1186/1476-0711-5-28 17121682 PMC1664579

[95] Di Azevedo MIN, Pires BC, Libonati H, Pinto PS, Cardoso Barbosa LF, Carvalho-Costa FA, Lilenbaum W. 2020. Extra-renal bovine leptospirosis: molecular characterization of the Leptospira interrogans Sejroe serogroup on the uterus of non-pregnant cows. Vet Microbiol 250:108869. doi:10.1016/j.vetmic.2020.108869 33010572

[96] R Core Team. 2024. R: a language and environment for statistical computing. R Foundation for Statistical Computing

[97] Oksanen J. 2024. Vegan: community ecology package

[98] Bartoń K. 2024. MuMIn: multi-model inference

