# Supplementary material for: Leptospira prevalence and lineages vary across land-use types due to shifts in small mammal communities
Source: Appl Environ Microbiol. 2026 Jan 30;92(2):e02061-25. doi: 10.1128/aem.02061-25 (PMC12915484; doi:10.1128/aem.02061-25)
Supplement: Supplemental material — Figures S1 to S3; Tables S1 to S4. [file aem.02061-25-s0001.docx]

**SUPPLEMENTARY MATERIALS**

Contents

[**Fig. S1.** Species composition comparisons between land use types. (A) Comparisons of the percent of native species and animal density (number captured/ effort), evenness, richness, and Shannon diversity of terrestrial small mammals. (B) Comparisons of species evenness, density (numbered captured), species richness, and Shannon diversity of bats between habitat types. Significant differences were found in the percentage of native species captured, density of animals, species richness and Shannon diversity of terrestrial mammals. Letters indicated significant differences (α=0.05) between habitat types based on Tukey’s honest significant differences. To plot all metrics together, each metric was scaled and centered, thus units on the y-axis are standard deviations. 1](#_Toc218689916)

[**Fig. S2.** All species version of Fig. 1A. 2](#_Toc218689917)

[**Fig. S4.** Interactive sankey diagram showing the patterns of land use gradients, small mammal host species and *Leptospira* species. All trapped small mammal species are represented. 4](#_Toc218689918)

[**Table S1.** Animal captures and *Leptospira* testing results by habitat type. Taxonomic groups endemic or native to Madagascar are designated with an asterisk. 5](#_Toc218689919)

[**Table S2.** Results of the Tukey post hoc test for pairwise comparisons species composition metrics of terrestrial small mammals between habitat types. 7](#_Toc218689920)

[**Table S3.** Full-model averaged coefficients and variable importance (AICc sum weight) for the binomial models of the probability of infection in terrestrial small mammals and bats. 10](#_Toc218689921)

[**Table S4.** *Leptospira* species by habitat type. 11](#_Toc218689922)


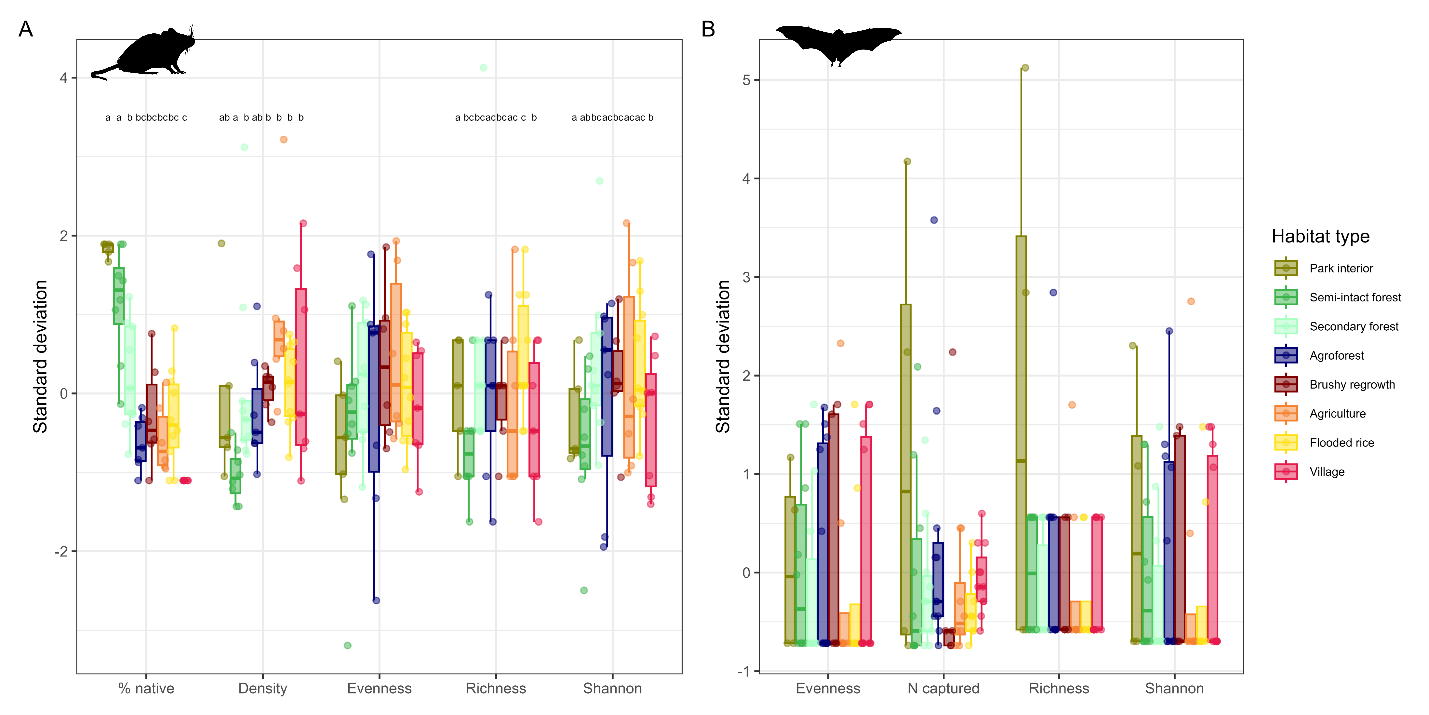


## **Fig. S1.** Species composition comparisons between land use types. (A) Comparisons of the percent of native species and animal density (number captured/ effort), evenness, richness, and Shannon diversity of terrestrial small mammals. (B) Comparisons of species evenness, density (numbered captured), species richness, and Shannon diversity of bats between habitat types. Significant differences were found in the percentage of native species captured, density of animals, species richness and Shannon diversity of terrestrial mammals. Letters indicated significant differences (α=0.05) between habitat types based on Tukey’s honest significant differences. To plot all metrics together, each metric was scaled and centered, thus units on the y-axis are standard deviations.


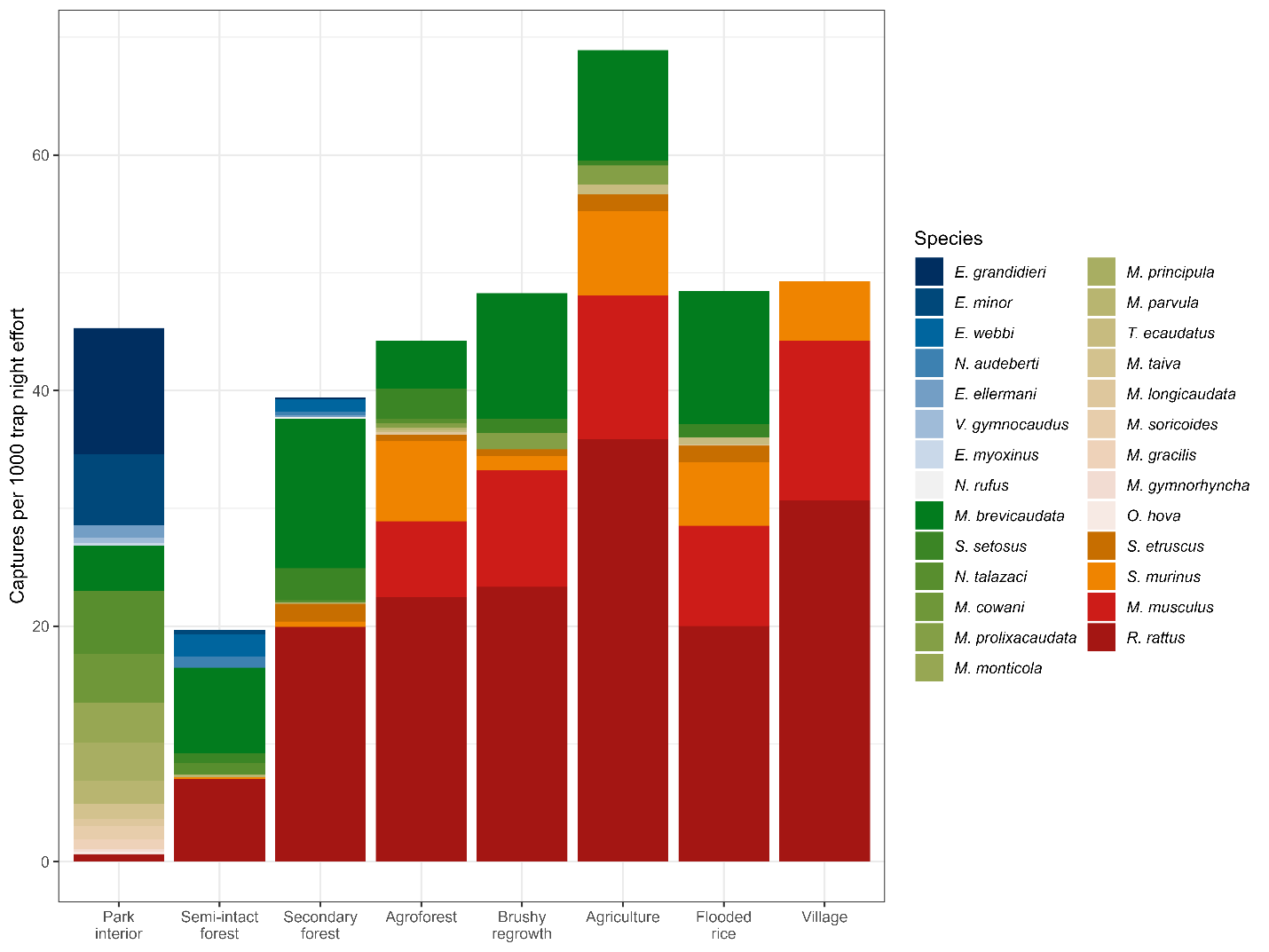


## **Fig. S2.** All species version of Fig. 1A.


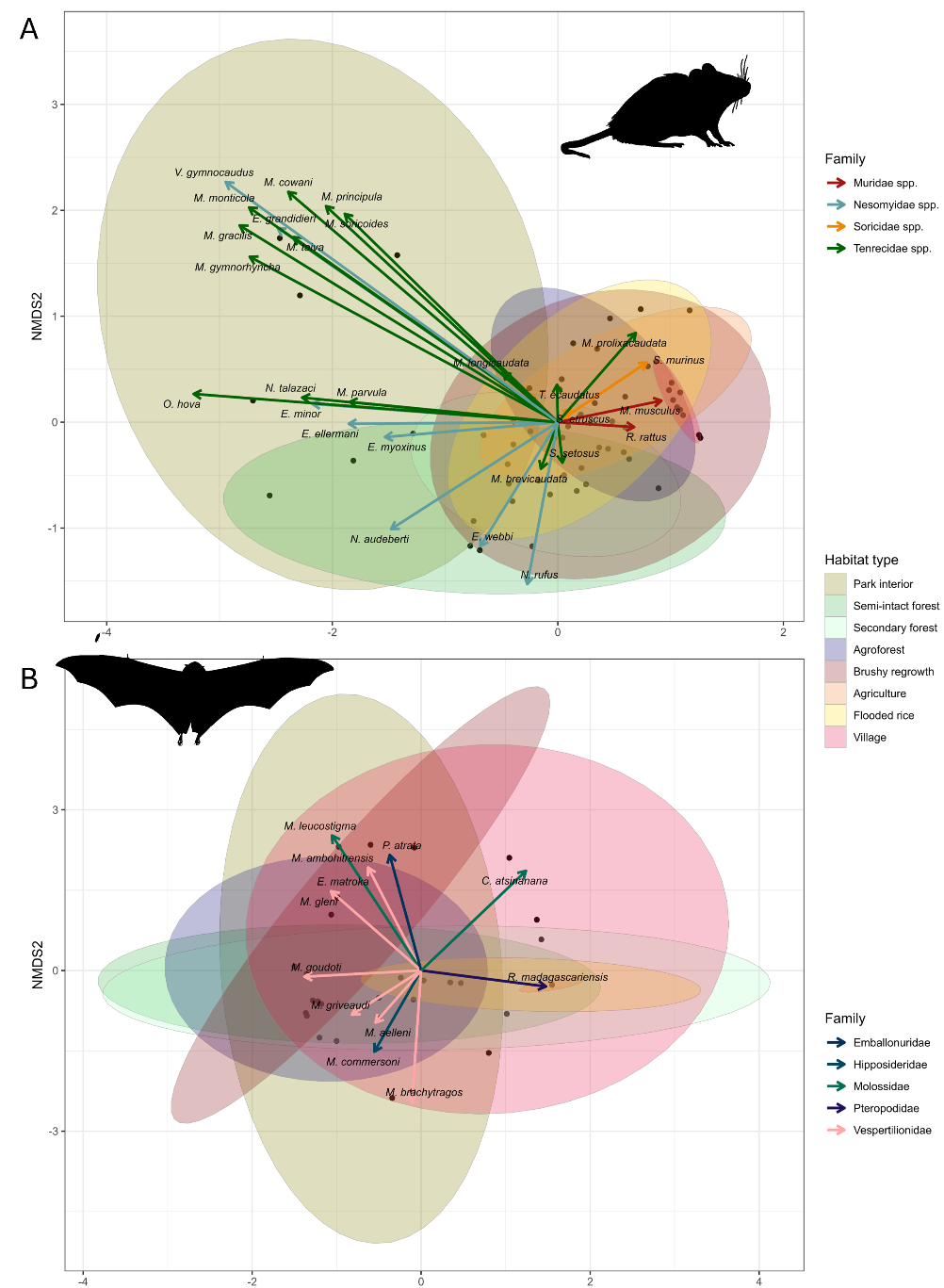


## **Fig. S3.** NMDS of (A) terrestrial mammals and (B) bats with arrows labeled by species. These plots complement Fig 1 panels C and D by displaying the respective community changes the pronounced separation of terrestrial mammals (A) between forested and non-forested habitat types and the lack of this contrast among (B) bats. The ellipses in the NMDS plots represent the 95% confidence intervals of the total population by habitat type (t-distributions) and the arrows indicate the strength and direction of the linear correlation between each species and the ordination scores. The arrows are colored by family and labeled by species.

See html file.

## **Fig. S4.** Interactive sankey diagram showing the patterns of land use gradients, small mammal host species and *Leptospira* species. All trapped small mammal species are represented.

## **Table S1.** Animal captures and *Leptospira* testing results by habitat type. Taxonomic groups endemic or native to Madagascar are designated with an asterisk.

| **Family** | **Species** | **Park interior** | **Semi-intact forest** | **Secondary forest** | **Agroforest** | **Brushy regrowth** | **Agriculture** | **Flooded rice** | **Village** | **TOTAL** |
| --- | --- | --- | --- | --- | --- | --- | --- | --- | --- | --- |
| **Terrestrial mammals [positive/tested; proportion positive (not tested)]** | | | | | | | | | | |
| Nesomyidae  subfamily  Nesomyinae* | *Eliurus grandidieri* | 10/50; 0.2 | - | 0/1; 0 | - | - | - | - | - | 10/51; 0.2 |
|  | *Eliurus minor* | 0/28; 0 | 0/3; 0 | - | - | - | - | - | - | 0/31; 0 |
|  | *Eliurus webbi* | - | 1/14; 0.07 | 1/7; 0.14 | - | - | - | - | - | 2/21; 0.1 |
|  | *Nesomys audeberti* | - | 1/7; 0.14 | 0/2; 0 | - | - | - | - | - | 1/9; 0.11 |
|  | *Eliurus ellermani* | 1/5; 0.2 | - | 0/1; 0 | - | - | - | - | - | 1/6; 0.17 |
|  | *Voalavo gymnocaudus* | 1/2; 0.5 | - | - | - | - | - | - | - | 1/2; 0.5 |
|  | *Eliurus myoxinus* | 0/1; 0 | - | - | - | - | - | - | - | 0/1; 0 |
|  | *Nesomys rufus* | - | - | 0/1; 0 | - | - | - | - | - | 0/1; 0 |
| Tenrecidae* | *Microgale brevicaudata* | 0/18; 0 | 7/54; 0.13 | 2/84; 0.02 | 0/21; 0 | 1/45; 0.02 (9) | 2/46; 0.04 | 8/98; 0.08 | - | 20/366; 0.05 (9) |
|  | *Setifer setosus* | - | 1/6; 0.17 | 0/18; 0 | 0/13; 0 | 0/5; 0 (1) | 0/2; 0 | 2/10; 0.2 | - | 3/54; 0.06 (1) |
|  | *Nesogale talazaci* | 1/25; 0.04 | 0/7; 0 | 0/1; 0 | 0/2; 0 | - | - | - | - | 1/35; 0.03 |
|  | *Microgale cowani* | 0/19; 0 | - | - | - | - | - | - | - | 0/19; 0 |
|  | *Microgale prolixacaudata* | - | - | - | 0/2; 0 | 0/7; 0 | 0/8; 0 | - | - | 0/17; 0 |
|  | *Microgale monticola* | 0/16; 0 | - | - | - | - | - | - | - | 0/16; 0 |
|  | *Microgale principula* | 0/15; 0 | - | - | - | - | - | - | - | 0/15; 0 |
|  | *Microgale parvula* | 0/9; 0 | 0/2; 0 | 0/1; 0 | 0/1; 0 | - | - | - | - | 0/13; 0 |
|  | *Tenrec ecaudatus* | - | - | - | 0/1; 0 | - | 0/4; 0 | 0/3; 0 (2) | - | 0/8; 0 (2) |
|  | *Microgale taiva* | 0/6; 0 | - | - | - | - | - | - | - | 0/6; 0 |
|  | *Microgale longicaudata* | 0/3; 0 | - | - | 0/1; 0 | - | - | 0/1; 0 | - | 0/5; 0 |
|  | *Microgale soricoides* | 0/5; 0 | - | - | - | - | - | - | - | 0/5; 0 |
|  | *Microgale gracilis* | 0/4; 0 | - | - | - | - | - | - | - | 0/4; 0 |
|  | *Microgale gymnorhyncha* | 0/1; 0 | - | - | - | - | - | - | - | 0/1; 0 |
|  | *Oryzorictes hova* | 0/1; 0 | - | - | - | - | - | - | - | 0/1; 0 |
| Soricidae | *Suncus etruscus* | - | - | 1/7; 0.14 (3) | 0/3; 0 | 0/3; 0 | 0/4; 0 (3) | 4/12; 0.33 | - | 5/29; 0.17 (6) |
|  | *Suncus murinus* | - | 0/1; 0 | 1/3; 0.33 | 0/35; 0 | 0/6; 0 | 2/35; 0.06 | 5/47; 0.11 | 1/21; 0.05 | 9/148; 0.06 |
| Muridae | *Mus musculus* | - | - | - | 12/33; 0.36 | 10/49; 0.2 (1) | 20/60; 0.33 | 40/74; 0.54 | 20/56; 0.36 | 102/272; 0.38 (1) |
|  | *Rattus rattus* | 0/3; 0 | 18/52; 0.35 | 12/131; 0.09 (1) | 17/116; 0.15 | 5/118; 0.04 | 18/176; 0.1 | 42/173; 0.24 (1) | 13/127; 0.1 | 125/896; 0.14 (2) |
| **TOTAL** | | 13/211; 0.06 | 28/146; 0.19 | 17/257; 0.07 (4) | 29/228; 0.13 | 16/233; 0.07 (11) | 42/335; 0.13 (3) | 101/418; 0.24 (3) | 34/204; 0.17 | **280/2032; 0.14 (21)** |
| **Bats [positive/tested; proportion positive (not tested)]** | | | | | | | | | | |
| Pteropodidae | *Rousettus madagascariensis** | 1/2; 0.5 | 0/0; 0 (3) | 0/5; 0 (9) | 4/15; 0.27 (17) | - | 5/17; 0.29 (11) | 5/18; 0.28 (6) | 1/1; 1 (21) | 16/58; 0.28 (67) |
| Emballonuridae | *Paremballonura atrata** | - | - | - | 0/9; 0 | 0/4; 0 | - | - | - | 0/13; 0 |
| Hipposideridae | *Macronycteris commersoni** | 1/3; 0.33 | 0/2; 0 | 0/1; 0 (1) | - | - | - | - | 0/1; 0 | 1/7; 0.14 (1) |
| Molossidae | *Chaerephon atsinanana** | - | - | - | - | - | - | - | 0/25; 0 | 0/25; 0 |
|  | *Mops leucostigma* | - | - | - | - | - | - | - | 0/0; 0 (6) | 0/0; 0 (6) |
| Vespertilionidae | *Miniopterus brachytragos** | - | - | - | - | - | - | 1/1; 1 | - | 1/1; 1 |
|  | *Miniopterus ambohitrensis** | 1/2; 0.5 | - | - | - | - | - | - | - | 1/2; 0.5 |
|  | *Laephotis matroka** | - | - | - | 0/1; 0 | - | - | - | - | 0/1; 0 |
|  | *Miniopterus aelleni* | 1/1; 1 | - | - | - | - | - | - | - | 1/1; 1 |
|  | *Miniopterus gleni** | - | - | - | 1/1; 1 | - | - | - | - | 1/1; 1 |
|  | *Myotis goudoti** | 28/45; 0.62 | 16/48; 0.33 | 14/28; 0.5 (5) | 11/21; 0.52 (17) | 0/1; 0 (14) | 0/1; 0 | 3/3; 1 | 4/6; 0.67 | 76/153; 0.5 (36) |
|  | *Miniopterus griveaudi* | 4/5; 0.8 | 0/4; 0 | 1/1; 1 | 1/5; 0.2 (1) | 0/0; 0 (8) | 1/2; 0.5 | 1/1; 1 | 1/1; 1 | 9/19; 0.47 (9) |
| **TOTAL** | | 36/58; 0.62 | 16/54; 0.3 (3) | 15/35; 0.43 (15) | 17/52; 0.33 (35) | 0/5; 0 (22) | 6/20; 0.3 (11) | 10/23; 0.43 (6) | 6/34; 0.18 (27) | **106/281; 0.38 (119)** |

## **Table S2.** Results of the Tukey post hoc test for pairwise comparisons species composition metrics of terrestrial small mammals between habitat types.

| **Comparison** | **Difference** | **lower** | **upper** | **p-value** |
| --- | --- | --- | --- | --- |
| **% native** | | | | |
| semi-intact forest-park interior | -0.2275 | -0.556 | 0.1009 | 0.3747 |
| **secondary forest-park interior** | **-0.539** | **-0.8604** | **-0.2177** | **0.0001** |
| **agroforest-park interior** | **-0.8203** | **-1.1577** | **-0.483** | **0** |
| **brushy regrowth-park interior** | **-0.7018** | **-1.0506** | **-0.3529** | **0** |
| **agriculture-park interior** | **-0.7994** | **-1.1482** | **-0.4505** | **0** |
| **flooded rice-park interior** | **-0.7105** | **-1.0261** | **-0.395** | **0** |
| **village-park interior** | **-0.9785** | **-1.3159** | **-0.6412** | **0** |
| **secondary forest-semi-intact forest** | **-0.3115** | **-0.5915** | **-0.0316** | **0.0194** |
| **agroforest-semi-intact forest** | **-0.5928** | **-0.891** | **-0.2946** | **0** |
| **brushy regrowth-semi-intact forest** | **-0.4743** | **-0.7854** | **-0.1631** | **0.0003** |
| **agriculture-semi-intact forest** | **-0.5719** | **-0.883** | **-0.2607** | **0** |
| **flooded rice-semi-intact forest** | **-0.483** | **-0.7563** | **-0.2097** | **0** |
| **village-semi-intact forest** | **-0.751** | **-1.0492** | **-0.4528** | **0** |
| *agroforest-secondary forest* | *-0.2813* | *-0.5716* | *0.0091* | *0.0639* |
| brushy regrowth-secondary forest | -0.1627 | -0.4664 | 0.1409 | 0.6903 |
| agriculture-secondary forest | -0.2603 | -0.564 | 0.0433 | 0.1431 |
| flooded rice-secondary forest | -0.1715 | -0.4362 | 0.0932 | 0.4608 |
| **village-secondary forest** | **-0.4395** | **-0.7298** | **-0.1491** | **0.0004** |
| brushy regrowth-agroforest | 0.1186 | -0.202 | 0.4391 | 0.9367 |
| agriculture-agroforest | 0.021 | -0.2996 | 0.3415 | 1 |
| flooded rice-agroforest | 0.1098 | -0.1741 | 0.3937 | 0.9212 |
| village-agroforest | -0.1582 | -0.4662 | 0.1498 | 0.7334 |
| agriculture-brushy regrowth | -0.0976 | -0.4302 | 0.235 | 0.9818 |
| flooded rice-brushy regrowth | -0.0088 | -0.3063 | 0.2887 | 1 |
| village-brushy regrowth | -0.2768 | -0.5973 | 0.0438 | 0.1374 |
| flooded rice-agriculture | 0.0888 | -0.2087 | 0.3863 | 0.9799 |
| village-agriculture | -0.1791 | -0.4997 | 0.1414 | 0.644 |
| village-flooded rice | -0.268 | -0.5519 | 0.0159 | 0.0772 |
| **Animal density** | | | | |
| semi-intact forest-park interior | *-0.771* | *-1.603* | *0.061* | *0.0878* |
| secondary forest-park interior | 0.1102 | -0.7039 | 0.9242 | 0.9999 |
| agroforest-park interior | -0.0268 | -0.8814 | 0.8277 | 1 |
| brushy regrowth-park interior | 0.2142 | -0.6695 | 1.0979 | 0.9941 |
| agriculture-park interior | 0.5554 | -0.3283 | 1.4391 | 0.5001 |
| flooded rice-park interior | 0.2086 | -0.5907 | 1.008 | 0.9908 |
| village-park interior | 0.1723 | -0.6822 | 1.0269 | 0.9981 |
| **secondary forest-semi-intact forest** | **0.8811** | **0.172** | **1.5903** | **0.0059** |
| agroforest-semi-intact forest | *0.7442* | *-0.0112* | *1.4995* | *0.0562* |
| **brushy regrowth-semi-intact forest** | **0.9852** | **0.197** | **1.7734** | **0.0055** |
| **agriculture-semi-intact forest** | **1.3264** | **0.5382** | **2.1145** | **0.0001** |
| **flooded rice-semi-intact forest** | **0.9796** | **0.2873** | **1.6719** | **0.0011** |
| **village-semi-intact forest** | **0.9433** | **0.188** | **1.6986** | **0.0056** |
| agroforest-secondary forest | -0.137 | -0.8725 | 0.5985 | 0.9989 |
| brushy regrowth-secondary forest | 0.1041 | -0.6651 | 0.8732 | 0.9999 |
| agriculture-secondary forest | 0.4452 | -0.3239 | 1.2144 | 0.6029 |
| flooded rice-secondary forest | 0.0985 | -0.5721 | 0.769 | 0.9998 |
| village-secondary forest | 0.0622 | -0.6733 | 0.7976 | 1 |
| brushy regrowth-agroforest | 0.2411 | -0.5709 | 1.053 | 0.9805 |
| agriculture-agroforest | 0.5822 | -0.2297 | 1.3942 | 0.3313 |
| flooded rice-agroforest | 0.2354 | -0.4838 | 0.9546 | 0.9666 |
| village-agroforest | 0.1991 | -0.581 | 0.9792 | 0.9919 |
| agriculture-brushy regrowth | 0.3412 | -0.5014 | 1.1837 | 0.9018 |
| flooded rice-brushy regrowth | -0.0056 | -0.7593 | 0.748 | 1 |
| village-brushy regrowth | -0.0419 | -0.8539 | 0.77 | 1 |
| flooded rice-agriculture | -0.3468 | -1.1004 | 0.4069 | 0.8267 |
| village-agriculture | -0.3831 | -1.195 | 0.4289 | 0.8079 |
| village-flooded rice | -0.0363 | -0.7555 | 0.6829 | 1 |
| **Species richness** | | | | |
| **semi-intact forest-park interior** | **-0.7358** | **-1.2704** | **-0.2011** | **0.0016** |
| **secondary forest-park interior** | **-0.6539** | **-1.177** | **-0.1308** | **0.0055** |
| agroforest-park interior | -0.4622 | -1.0113 | 0.087 | 0.1589 |
| **brushy regrowth-park interior** | **-0.7326** | **-1.3005** | **-0.1647** | **0.0038** |
| agriculture-park interior | -0.4252 | -0.9931 | 0.1427 | 0.2795 |
| **flooded rice-park interior** | **-0.5179** | **-1.0315** | **-0.0042** | **0.0469** |
| **village-park interior** | **-1.0747** | **-1.6238** | **-0.5255** | **0** |
| secondary forest-semi-intact forest | 0.0819 | -0.3738 | 0.5376 | 0.9991 |
| agroforest-semi-intact forest | 0.2736 | -0.2118 | 0.759 | 0.6342 |
| brushy regrowth-semi-intact forest | 0.0032 | -0.5033 | 0.5097 | 1 |
| agriculture-semi-intact forest | 0.3106 | -0.1959 | 0.8171 | 0.5314 |
| flooded rice-semi-intact forest | 0.2179 | -0.2269 | 0.6628 | 0.777 |
| village-semi-intact forest | -0.3389 | -0.8243 | 0.1465 | 0.3647 |
| agroforest-secondary forest | 0.1917 | -0.2809 | 0.6643 | 0.9009 |
| brushy regrowth-secondary forest | -0.0787 | -0.573 | 0.4156 | 0.9996 |
| agriculture-secondary forest | 0.2287 | -0.2656 | 0.723 | 0.8226 |
| flooded rice-secondary forest | 0.136 | -0.2949 | 0.5669 | 0.9726 |
| village-secondary forest | -0.4208 | -0.8934 | 0.0519 | 0.1141 |
| brushy regrowth-agroforest | -0.2704 | -0.7922 | 0.2514 | 0.7247 |
| agriculture-agroforest | 0.037 | -0.4848 | 0.5588 | 1 |
| flooded rice-agroforest | -0.0557 | -0.5179 | 0.4065 | 0.9999 |
| **village-agroforest** | **-0.6125** | **-1.1138** | **-0.1112** | **0.0072** |
| agriculture-brushy regrowth | 0.3074 | -0.234 | 0.8489 | 0.6258 |
| flooded rice-brushy regrowth | 0.2147 | -0.2696 | 0.699 | 0.8519 |
| village-brushy regrowth | -0.3421 | -0.8638 | 0.1797 | 0.4455 |
| flooded rice-agriculture | -0.0927 | -0.577 | 0.3916 | 0.9987 |
| **village-agriculture** | **-0.6495** | **-1.1713** | **-0.1277** | **0.0058** |
| **village-flooded rice** | **-0.5568** | **-1.019** | **-0.0946** | **0.0085** |
| **Shannon diversity** | | | | |
| semi-intact forest-park interior | *-0.4448* | *-0.9092* | *0.0196* | *0.0696* |
| **secondary forest-park interior** | **-0.5291** | **-0.9835** | **-0.0747** | **0.0123** |
| agroforest-park interior | -0.3078 | -0.7849 | 0.1692 | 0.4659 |
| **brushy regrowth-park interior** | **-0.5246** | **-1.0179** | **-0.0313** | **0.0297** |
| agriculture-park interior | -0.3366 | -0.8299 | 0.1567 | 0.3937 |
| flooded rice-park interior | -0.3633 | -0.8095 | 0.0829 | 0.1895 |
| village-park interior | -0.8207 | -1.2977 | -0.3437 | 0 |
| secondary forest-semi-intact forest | -0.0843 | -0.4801 | 0.3116 | 0.9974 |
| agroforest-semi-intact forest | 0.1369 | -0.2847 | 0.5586 | 0.968 |
| brushy regrowth-semi-intact forest | -0.0798 | -0.5198 | 0.3601 | 0.999 |
| agriculture-semi-intact forest | 0.1081 | -0.3318 | 0.5481 | 0.9936 |
| flooded rice-semi-intact forest | 0.0815 | -0.3049 | 0.4679 | 0.9975 |
| village-semi-intact forest | -0.3759 | -0.7976 | 0.0457 | 0.113 |
| agroforest-secondary forest | 0.2212 | -0.1893 | 0.6318 | 0.6845 |
| brushy regrowth-secondary forest | 0.0044 | -0.4249 | 0.4338 | 1 |
| agriculture-secondary forest | 0.1924 | -0.2369 | 0.6218 | 0.8449 |
| flooded rice-secondary forest | 0.1658 | -0.2085 | 0.5401 | 0.8526 |
| village-secondary forest | -0.2916 | -0.7022 | 0.1189 | 0.3429 |
| brushy regrowth-agroforest | -0.2168 | -0.67 | 0.2365 | 0.797 |
| agriculture-agroforest | -0.0288 | -0.482 | 0.4244 | 1 |
| flooded rice-agroforest | -0.0554 | -0.4569 | 0.346 | 0.9998 |
| **village-agroforest** | **-0.5129** | **-0.9483** | **-0.0774** | **0.0109** |
| agriculture-brushy regrowth | 0.188 | -0.2824 | 0.6583 | 0.9076 |
| flooded rice-brushy regrowth | 0.1613 | -0.2593 | 0.582 | 0.9243 |
| village-brushy regrowth | -0.2961 | -0.7493 | 0.1571 | 0.4501 |
| flooded rice-agriculture | -0.0266 | -0.4473 | 0.394 | 1 |
| **village-agriculture** | **-0.4841** | **-0.9373** | **-0.0308** | **0.0286** |
| **village-flooded rice** | **-0.4574** | **-0.8589** | **-0.056** | **0.0154** |

## **Table S3.** Full-model averaged coefficients and variable importance (AICc sum weight) for the binomial models of the probability of infection in terrestrial small mammals and bats.

| **Effect (reference category)** | **Estimate** | **Std. Error** | **95% CI** | **50% CI** | **Importance (N containing models)** |
| --- | --- | --- | --- | --- | --- |
| **Terrestrial small mammals** | | | | | |
| **Habitat type** (park interior) |  | | | | |
| Semi-intact forest | 0.42 | 0.65 | -0.86, 1.71 | -0.01, 0.86 | 1 (4) |
| Secondary forest | -0.54 | 0.58 | -1.67, 2.60 | -0.92, -0.15 |  |
| Agroforest | 0.42 | 0.53 | -0.61, 3.45 | 0.07, 0.77 |  |
| Brushy regrowth | -0.19 | 0.56 | -1.28, 4.90 | -0.56, 0.18 |  |
| Agriculture | 0.28 | 0.52 | -0.75, 5.30 | -0.07, 0.62 |  |
| Flooded rice | 0.78 | 0.52 | -0.23, 6.79 | 0.44, 1.13 |  |
| Village | -0.08 | 0.54 | -1.15, 7.98 | -0.44, 0.28 |  |
| **Year** (2017) |  | | | | 1 (4) |
| 2018 | 0.83 | 0.26 | 0.31, 9.34 | 0.65, 1.00 |  |
| 2019 | 0.32 | 0.27 | -0.20, 10.85 | 0.15, 0.50 |  |
| 2020 | -0.77 | 0.27 | -1.30, 11.25 | -0.95, -0.60 |  |
| 2021 | -0.55 | 0.43 | -1.40, 12.29 | -0.84, -0.27 |  |
| Sqrt (**Richness**) | 0.09 | 0.20 | -0.31, 13.49 | -0.05, 0.23 | 0.22 (1) |
| Log (**Density**) | 0.28 | 0.30 | -0.32, 8.87 | 0.07, 0.48 | 0.56 (2) |
| **Season** (Warm wet) |  | | | | 0.15 (1) |
| Cool wet | -0.04 | 0.15 | -0.34, 14.26 | -0.14, 0.06 |  |
| Hot dry | -0.09 | 0.26 | -0.60, 15.41 | -0.27, 0.08 |  |
| **Bats** | | | | | |
| **Season** (Cool wet) |  | | | | 1 (3) |
| Hot dry | 1.08 | 0.34 | 0.42, 16.75 | 0.86, 1.31 |  |
| **Richness** | 0.03 | 0.06 | -0.08, 17.14 | -0.01, 0.07 | 0.33 (1) |
| **N captured** | 0.00 | 0.01 | -0.01, 18.02 | 0.00, 0.01 | 0.28 (1) |

## **Table S4.** *Leptospira* species by habitat type.

| **Species** | **Park interior** | **Semi-intact forest** | **Secondary forest** | **Agroforest** | **Brushy regrowth** | **Agriculture** | **Flooded rice** | **Village** |
| --- | --- | --- | --- | --- | --- | --- | --- | --- |
| L. borgpetersenii | 13; 0.27 | 14; 0.32 | 11; 0.34 | 9; 0.2 | - | - | 3; 0.03 | 3; 0.07 |
| L. mayottensis | 2; 0.04 | - | - | - | - | - | - | - |
| L. interrogans | - | - | 7; 0.22 | 2; 0.04 | - | 3; 0.06 | 15; 0.14 | 5; 0.12 |
| L. kirschneri | - | - | 2; 0.06 | 3; 0.07 | 5; 0.31 | 7; 0.15 | 19; 0.17 | 7; 0.17 |
| L. sp. a | - | - | - | 1; 0.02 | - | 1; 0.02 | - | - |
| L. sp. c | - | - | - | - | - | 1; 0.02 | 1; 0.01 | - |
| L. sp. b | - | - | - | - | - | - | 2; 0.02 | - |
| Unknown | 34; 0.69 | 30; 0.68 | 12; 0.38 | 31; 0.67 | 11; 0.69 | 36; 0.75 | 71; 0.64 | 25; 0.62 |
